# Radiotherapy, PARP Inhibition, and Immune-Checkpoint Blockade: A Triad to Overcome the Double-Edged Effects of Each Single Player

**DOI:** 10.3390/cancers15041093

**Published:** 2023-02-08

**Authors:** Maria Manuela Rosado, Claudio Pioli

**Affiliations:** 1 Consultant in Immunology, 00125 Rome, Italy; 2 Department of Clinical Internal Sciences, Anesthesiology and Cardiovascular Sciences, Sapienza University of Rome, 00161 Rome, Italy; 3 Laboratory of Biomedical Technologies, Division of Health Protection Technologies, ENEA, 00123 Rome, Italy

**Keywords:** radiotherapy, immunotherapy, PARP inhibitors, tumor immunity, combined therapies, cancer immunology, immune checkpoints

## Abstract

**Simple Summary:**

While radiotherapy remains a key therapy for many cancers, in recent years, DNA repair inhibitors, particularly PARP inhibitors, and immunotherapy, specifically immune-checkpoint inhibitors, have progressively shown great therapeutic potential in several experimental and clinical settings. In the present review, we discuss the beneficial and disadvantageous effects of each approach and how these three therapies can synergize, overcoming single-therapy limitations.

**Abstract:**

Radiotherapy and, more recently, PARP inhibitors (PARPis) and immune-checkpoint inhibitors represent effective tools in cancer therapy. Radiotherapy exerts its effects not only by damaging DNA and inducing tumor cell death, but also stimulating anti-tumor immune responses. PARPis are known to exert their therapeutic effects by inhibiting DNA repair, and they may be used in combination with radiotherapy. Both radiotherapy and PARPis modulate inflammatory signals and stimulate type I IFN (IFN-I)-dependent immune activation. However, they can also support the development of an immunosuppressive tumor environment and upregulate PD-L1 expression on tumor cells. When provided as monotherapy, immune-checkpoint inhibitors (mainly antibodies to CTLA-4 and the PD-1/PD-L1 axis) result particularly effective only in immunogenic tumors. Combinations of immunotherapy with therapies that favor priming of the immune response to tumor-associated antigens are, therefore, suitable strategies. The widely explored association of radiotherapy and immunotherapy has confirmed this benefit for several cancers. Association with PARPis has also been investigated in clinical trials. Immunotherapy counteracts the immunosuppressive effects of radiotherapy and/or PARPis and synergies with their immunological effects, promoting and unleashing immune responses toward primary and metastatic lesions (abscopal effect). Here, we discuss the beneficial and counterproductive effects of each therapy and how they can synergize to overcome single-therapy limitations.

## 1. Introduction

Cancer represents the second leading cause of death worldwide with 10 million deaths and 24 million cases in 2019, with an increasing global burden and a huge impact on life quality [1]. Where available, cancer treatments include surgery, radiotherapy, chemotherapy, immunotherapy, and targeted therapy. Along the years, radiotherapy (RT) has largely contributed to better disease control, reducing nontargeted tissue toxicity and improving overall survival through the use of conformal and intensity-modulated RT or charged particles. However, the appearance of local recurrences and metastases after RT has underlined the need for additional therapies. The last decade saw the progressive approval of monoclonal antibodies targeting negative costimulatory immune receptors known as immune-checkpoint inhibitors (ICIs), which achieved considerable therapeutic success in a certain number of advanced cancers. A better understanding of the effects of RT dose and fractionation allowed uncovering the immunological effects of RT and developing combined therapies between RT and immunotherapy. With the approval of the first PARP inhibitor (PARPi) in 2014, new therapeutic tools targeting DNA repair mechanisms have progressively become available, and synergies with RT have been explored. In the present review, we discuss the beneficial and detrimental effects of each single therapy, namely, RT, PARPis, and ICIs, and how they can synergize to overcome single-therapy limitations.

## 2. Radiotherapy

Radiotherapy plays a major role in the treatment of a wide range of malignancies. Around 60–70% of patients undergo treatments, mostly with photon therapy (X- or γ-rays), in addition to others with heavy ions and protons. Although radiotherapy has been in use for many decades, it is during this century that new underlying mechanisms of action, the relevance of fractionation/hypo-fractionation, and synergies with other therapies have been better understood and, thus, exploited [2].

Ionizing radiations (IRs) induce DNA damage with the number, distribution, variety, and severity of lesions depending on the quality of radiation, dose, fractionation, cell physiological status, and tumor microenvironment (TME) (including oxygen availability). DNA damage is induced by direct energy deposition or indirectly through the generation of highly reactive free radicals. Lesions induced by a single ionizing trajectory localize within short distances (nanometers); these clustered lesions are a typical signature of IR-damaged DNA [3,4]. The large number of clustered DNA lesions, including multiple double-strand breaks, generated by IR are hardly fixed by the DNA repair mechanisms and lead to cell-cycle arrest and/or cell death [5].

While radiation toxicity has been considered the (main) mechanism of action in radiotherapy, its collateral tissue damage has urged more favorable ratios between the dose adsorbed by the targeted tumor and the normal tissues. Both three-dimensional conformal and intensity-modulated RT reduced nontargeted tissue toxicity and improved overall survival compared with two-dimensional RT, although with some contrasting conclusions [6,7]. A better dose distribution on the targeted tissue compared with photon RT can be obtained with charged particles. While traveling through tissues, protons and carbon ions, the most used charged particles for RT, release energy according to a typical curve ending with a pronounced peak (Bragg peak). At the Bragg peak, the majority of the energy is released (with a tiny lateral scatter) and a massive ionization of the surrounding matter occurs. Tissues lying beyond the Bragg peak are, therefore, spared. Superimposition of multiple Bragg peaks spanning the tumor volume improves disease control [8]. Better results, in terms of overall survival and disease control, are obtained with carbon ion compared with both proton and photon RT, due to greater linear energy transfer and superior relative biological effectiveness, especially in tumors requiring higher RT doses and developing in critical locations [9,10].

## 3. Immuno-Stimulating Effects of Radiotherapy

During the last two decades, it has become progressively evident that IRs do not exert their effects exclusively by direct killing of tumor cells. Radiotherapy has important immunological effects by inducing the expression of IFNs, as well as other cytokines and chemokines, the release of tumor-associated antigens (TAA), immunogenic cell death (ICD), and changes in the TME [11,12].

IR-induced leakage of nuclear and mitochondrial DNA into the cytosol activates the cyclic GMP–AMP synthase (cGAS)/stimulator of interferon genes (STING) pathway. cGAS/STING signaling, a pathway normally involved in antiviral responses, results in the expression of type I IFN (IFN-I) in irradiated cells and sustains the antitumor immune response [13,14]. IFN-I, together with other signals, promotes recruitment and activation of dendritic cells (DCs), which in turn activates CD8 cells to perform T-cell killing, a process essential for tumor reduction [15]. Noteworthily, experiments comparing equivalent doses of photon, proton, and carbon ion IRs showed that, despite differences at early timepoints, all these radiotherapeutic agents induced a similar gene expression signature in exposed tumor cells involving the activation of the GAS/STING pathway and STAT1-dependent responses [16].

**Figure 1:**
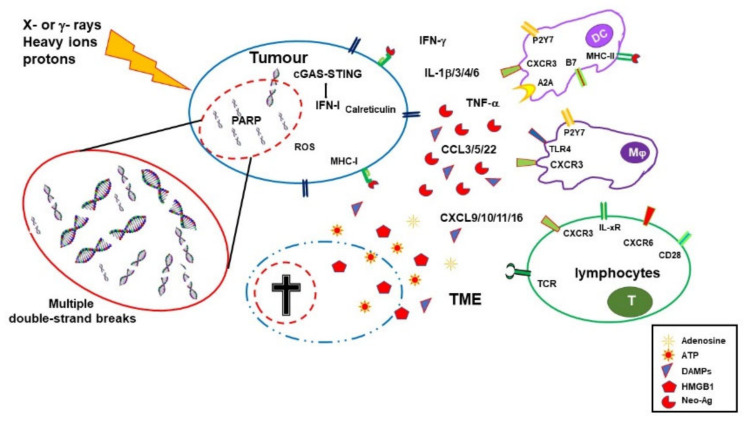
Immuno-stimulating effects of radiotherapy. Ionizing radiations (IRs) induce DNA damage, oxidative stress, and cell death. Dying cells release HMGB1 and ATP, and then expose calreticulin on their cell surface, all these molecules being features of immunogenic cell death. Damaged DNA activates the cGAS/STING pathway, leading to IFN-I expression, upregulation of MHC I expression, and improved antigen presentation in surviving cells. IRs also alter the tumor microenvironment (TME) by inducing the expression of several cytokines and chemokines with consequent recruitment of leukocytes. Tumor cells might accumulate mutations and express neoantigens which are taken up by dendritic cells (DCs) that, in the presence of the inflammatory stimuli, mature and migrate to the draining lymph node where they prime tumor antigen-specific CD4 and CD8 T cells.

In addition to the upregulation of IFN-I, RT induces the expression of several cytokines and chemokines, consequently orchestrating recruitment and activation (or suppression, see below) of several leukocyte populations into the tumor site. RT-induced cytokines include IFNγ, IL1β, TNFα, IL-3, IL-4, IL-6, and TGFβ [17]. Cytokines and chemokines are known to mutually regulate their expression. RT-induced IL1β expression, for instance, upregulates CCL2 production and, consequently, sustains the recruitment of CCR6^+^ monocytes and T cells [18]. Upregulation of several chemokines, including CCL3, CCL5, CCL22, CXCL9, CXCL10, and CXCL11, has been described to play different roles with effects depending on tumor type and other TME factors [19,20]. CXCL16 is also upregulated by IRs in both mouse and human breast cancer cells, representing a major factor in driving CXCR6-expressing Th1 and CD8 T cells to the tumor site [21]. In fact, RT sustains CD8 T-cell recruitment into the tumor by inducing the expression of several factors, including IFNα, CXCL9, CXCL10, and CXCL11, as well as promoting extravasation by upregulating ICAM-1 and E-selectin on endothelial cells [17,22,23]. It is of note that several of the abovementioned cytokines are still upregulated in irradiated tissues and peripheral blood for several weeks after treatment [19] (Figure 1).

RT-injured tumor and tumor-infiltrating cells release intracellular molecules known as damage-associated molecular patterns (DAMPs), or alarmins, including high-mobility group box 1 (HMGB1), ATP, and calreticulin. DAMPs are released through both passive mechanisms, due to cell damage-associated leakage, and different active processes, depending on the stressing stimulus, which includes RT-induced reactive oxygen species (ROS) generation. Through specific receptors, DAMPs are recognized as danger signals by immune and nonimmune cells, resulting in inflammatory response, with the release of chemotactic factors, upregulation of adhesion molecules, and leukocyte recruitment and activation. Danger signals, therefore, generate the immunogenic context promoting immune responses toward TAA released by RT-damaged cells in the TME [24].

HMGB1, passively released by dying cells or actively secreted by inflammation-stimulated cells, is recognized by Toll-like receptor 4 (TLR4) and by the receptor for advanced glycation end-products (RAGE), both expressed on several cells, including macrophages and dendritic cells. TLR4 and RAGE engagement by HMGB1 leads to NF-κB activation and expression of proinflammatory cytokines, release of chemotactic factors, and recruitment and activation of leukocytes [25]. Noteworthily, TLR4 polymorphisms that reduce its ability to bind HMGB1 reduce tumor antigen processing and presentation by dendritic cells. Indeed, after radiotherapy, breast cancer patients with a compromised HMGB1 engagement by TLR4 undergo relapses more rapidly than patients bearing normal TLR4 alleles [26].

The concentration of ATP in the extracellular space modulates different functions including cell differentiation, proliferation, adhesion, and death. Any type of cell death induces secretion or release of ATP, although the involved mechanisms depend on the type of death stimulus and the apoptotic stage. Extracellular ATP is perceived as a “find me” signal which drives macrophages to the dying cells through P2Y2 receptors [24]. However, when present at higher concentrations, the ATP can also be recognized by the purinergic P2X7 receptors on dendritic cells and activate the NALP3-inflammasome pathway, thus acting as a danger signal and inducing immunogenic responses. ATP-stimulated dendritic cells produce IL-1 and IL-18, which synergize with IFNγ in the induction of tumor-specific CD8 T cells. The relevance of this pathway in contributing to the immune response against tumor cells is sustained by the finding that patients with breast cancer bearing a mutated P2X7 receptor progressed to metastatic disease more quickly than patients with a functional P2X7 receptor [27] (Figure 1).

Calreticulin is a molecule mostly localized in the endoplasmic reticulum, playing several immune roles including assembly of MHC I molecules and loading of peptides on the MHC I groove. Calreticulin is involved in cell signaling, Ca^2+^ homeostasis, and cell migration and proliferation [28]. Production of ROS and reactive nitrogen species (RNS), induced by photon or proton radiotherapy, leads to endoplasmic reticulum stress and calreticulin exposure on the external cell membrane [29,30]. Exposed calreticulin represents an “eat me” signal for dendritic cells and macrophages, which leads to natural killer cell and neutrophil recruitment to the tumor site [31,32]. Indeed, induction of immunogenic cell death by cancer therapies relies on the generation of reactive oxygen species and/or of endoplasmic reticulum stress [24].

Photon radiotherapy increases the expression of MHC class I molecules on tumor cells, a finding that was also observed, more recently, using protons [30]. RT induces the expression of novel proteins and neoantigens, as well as enhances protein degradation and the generation of additional peptides, which are presented to CD8 T cells in association with MHC class I molecules. These two properties may cooperate to increase antigen presentation and activation of tumor specific CD8 immune responses. Indeed, dendritic cells take up TAA and, in the presence of inflammatory stimuli, mature and migrate to draining lymph nodes where they prime TAA-specific naïve T cells [33,34]. Engulfment of cancer cells by dendritic cells leads to cross-presentation of TAA and stimulation of TAA-specific CD8 T cells [35]. Ablative radiotherapy induces CD8 T-cell priming by DC in draining lymph nodes, resulting in T-cell-dependent tumor control, as shown in animal models [36].

Radiotherapy, therefore, by inducing the release of DAMPs, the expression of cytokines and chemokines, cell death, and the release/expression of TAA, has the potential to create an inflammatory/immunogenic context where innate and TAA-specific adaptive immune cells could be activated and generate an antitumor immune response (Figure 1), thus providing the rationale for radioimmunological synergic therapies [37,38].

## 4. Immuno-Depressing Effects of Radiotherapy

RT has been known for a long time to induce immunosuppressive effects through a toxic action. Leukopenia is one of the most frequent effects of RT. When the area of exposure includes bone marrow cells, a long time is required to fully recover the hematopoietic damage both in clinic and in experimental settings [39,40,41]. However, leukocytes display different grades of susceptibility to the effects of IRs, depending on cell type, activation status, and cell-cycle phase. Myeloid cells, including monocytes/macrophages, dendritic cells, and myeloid-derived suppressor cells (MDSCs), are more resistant to IR than lymphocytes and NK cells, probably for their reduced proliferative rate compared with lymphoid cells [42]. Within the myeloid lineage, circulating monocytes are more susceptible than tissue macrophages [43]. Moreover, M2-polarized macrophages, which play an unfavorable role in tumor immunity, have been shown to be more resistant to IRs than M1-polarized macrophages, both in vivo and in vitro and under both normoxia and hypoxia [44,45].

Lymphocytes are more susceptible than myeloid cells, and they undergo apoptosis during the interphase if irradiated. B cells have been shown to be particularly susceptible to radiations [42]. T cells are quite heterogeneous in their susceptibility, with activated T cells being more resistant than resting cells and CD4 being more resistant than CD8 T cells. Tumor-associated and tissue-resident memory T cells were described to be more resistant than naïve T cells, probably for their pre-activated status and for the protective effects of the TGFβ often present in the TME [46]. Within the CD4 T-cell population, Foxp3-expressing regulatory T cells appear to be more resistant. Upon radiation, the percentage of Treg cells is increased within the tumor site compared with CD8 and non-Treg CD4 cells [47,48]. However, this can also occur due to de novo regulatory T-cell recruitment into the tumor [49]. Lymphopenia is also induced by irradiation of lymphatic structure, as it is the case of elective lymph node irradiation, where the majority of the cells are T cells [50]. Furthermore, it should be considered that local highly repeated irradiation can expose, according to the specific tumor site, up to almost all circulating peripheral blood cells, leading to leukopenia [51]. RT, therefore, not only reduces the number of leukocytes but it can also alter the relative composition of tumor-infiltrating leukocytes in favor of more suppressive cells.

**Figure 2:**
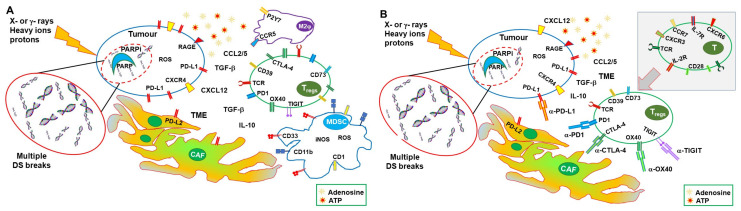
Correcting the immune suppressive effects of RT and PARPis into a hot stimulating TME by targeting immune checkpoints. (**A**) RT induces immune-suppressive effects through several mechanisms other than simple cell toxicity. RT upregulates the expression of chemokines (CCL2 and CCL5) and cytokines (IL-10 and TGFβ), which sustain recruitment of MDSC and Treg cells, polarization of M2 macrophages and Treg cells, and inhibitory effects on the immune response. RT also induces the expression of PD-L1 in tumor and immune cells, as well as of PD-L2 in cancer-associated fibroblasts (CAF). Furthermore, PARPis sustain regulatory T cell (Treg) recruitment and PD-L1 upregulation. A suppressive TME is also favored by an excessive release of ATP, which is rapidly degraded to adenosine by CD39 and CD73, compromising the antitumor immune response. (**B**) The addition of ICIs to therapy can sustain the beneficial effects of RT and PARPis, leading to a synergic immune stimulation. A hot immunogenic TME allows DC to take up tumor-associated antigens (TAA) and migrate to draining lymph nodes, where anti-CTLA-4 ICIs, by lowering the threshold for activation, sustain the generation of effector CD4 and CD8 T cells. Effector T cells can migrate (upper corner) to the tumor site and exert their functions. At the tumor site, anti-PD-1 and/or anti-PD-L1 ICIs prevent the inhibition of the effector T cells by PD-L1-expressing tumor (and nontumor) cells. Both anti-CTLA-4 and anti-PD-1 ICIs can contrast the action of Treg cells, unleashing the antitumor immune response.

The Immune suppressive effects of IRs are also induced by prolonged and/or intense activation of signals normally associated with immune activation. Expression of inhibitory cytokines, recruitment of T and myeloid regulatory cells, and an immune-suppressive TME are associated with repeated irradiation [52]. Irradiation-induced chronic expression of IFN-I leads to the upregulation of PD-L1, as shown in tumor cell lines and in tumor-infiltrating macrophages [53,54]. STING-sustained IFN-I production also results in increased expression of CCL2 and recruitment of monocytic MDSC and Treg cells [55,56]. In addition, RT may induce the expression of CCL5, which, in synergy with CCL2, increases the recruitment of immunosuppressive monocytes, dampening the therapeutic effects of RT [18]. Furthermore, RT-generated ROS enhances the production and activation of TGFβ from several tumor-infiltrating leukocytes and other tumor-associated cells [57], which sustains recruitment of MDSC, polarization of M2 macrophages and Treg cells, and differentiation of cancer-associated fibroblasts (CAFs) [58,59]. CAFs, in turn, further raise recruitment and survival of Treg cells through the secretion of CXCL12 and expression of PD-L2, sustaining a positive loop of immunosuppression [60] (Figure 2A).

Additionally, DAMPs can be double-edged swords in the immune response against tumors [61]. Indeed, nuclear HMGB1 is involved in DNA repair, autophagy, and tumor radioresistance [62], while extracellular HMGB1 can stimulate tumor cell proliferation through the RAGE–Erk/p38 pathway [63]. RT-induced release of HMGB1 can also induce the recruitment of MDSCs, which, as previously described, contribute to immune suppression [64].

On the other hand, ATP is enzymatically hydrolyzed by CD39 and CD73 to adenosine, which is a strong anti-inflammatory mediator and can compromise the antitumor immune response [65]. High levels of ATP can, therefore, contribute to generate an adenosine-rich tumor microenvironment and favor tumor immune escape. Noteworthy, the adenosine A2A receptor deficiency increases tumor rejection [66], while CD73 targeting reduces tumorigenesis [67]. Moreover, CD39 overexpression compromises the immunogenicity of cell death, probably by both quickly removing the stimulatory ATP molecule and generating the suppressive adenosine mediator [68].

Thus, RT can induce immune-suppressive effects through several mechanisms other than simple toxicity (Figure 2A), and immune-stimulating effects, with the balance depending on tumor type, prevailing factors, and the TME. Synergy with other therapies can alter the equilibrium in favor of an effective immune response.

## 5. PARP Inhibitors

PARP-1, the most abundant member of the poly(ADP-ribose) (PAR) polymerase (PARP) family, more recently defined as diphtheria toxin-like ADP-ribosyltransferases (ARTDs), accounts for the majority of PARylation activity and has a high DNA damage-sensing ability [69]. Free DNA ends activate PARP-1, which highly PARylates itself and detaches from chromatin. Indeed, addition of PARs radically changes the electric charge of the targeted molecule, rendering it highly negative. As a consequence, PARylated proteins are electrostatically repulsed by the DNA, a mechanism involved in chromatin accessibility to DNA repair enzymes (and to DNA transcription and replication regulators). PARP-1 also generates large amounts of PARs that work as scaffolds recruiting DNA repair enzymes to the lesion site, including XRCC1 [70]. PARP-1 plays a central role in orchestrating responses to genotoxic stress and represents a critical enzyme in single-strand break and alternative end-joining repair [71,72]. However, recent studies also indicated that PARP-1 plays a role in double-strand break (DSB) repair mechanisms, including homologous recombination and classical nonhomologous end-joining (c-NHEJ) [73,74].

Following a long period of preclinical and clinical studies, PARP inhibitors (PARPis) reached wide clinical use with the approval of olaparib (AZD-2281) in 2014 and later on of niraparib (MK-4827), rucaparib (AG-014699), talazoparib (BMN673), and veliparib (ABT888) for treatment of ovarian, breast, prostate, and pancreatic cancer [75,76]. PARPis are the first clinically approved drugs exploiting synthetic lethality; that is, they target a function specifically vital in mutation-bearing cancer cells [77,78]. PARPis were shown to be lethal in homologous recombination (HR)-deficient BRCA1/BRCA2-mutated cancers, likely because collapsed replication forks are no longer repaired [79,80]. However, recent preclinical and early clinical studies also sustained the use of PARPis in other molecular subsets of cancer, including cancers with high replication stress [81].

All clinically approved PARPis share a nicotinamide-based moiety that inhibits PARP-1 enzymatic activity by competing for binding to the catalytic site with NAD. PARPis prevent PARP-1 auto-PARylation and its consequent removal from chromatin and DNA lesions. This effect, termed PARP trapping, is currently the preferred interpretative model of the PARPis mechanism of action. Indeed, cytotoxicity due to PARP correlates with the ability to trap PARP on DNA lesions and is more cytotoxic than gene deletion. PARP trapping leads to replication fork collapse during the S phase and consequent cell death [82,83].

The wide clinical use has revealed that tumor clones resistant to PARPis may appear during therapy compromising clinical outcome. Tumors escape PARPi effects either by restoring HR or by protecting the DNA replication fork [84]. Recovery of HR functions, which can happen through a higher compensating expression of the functional allele, loss of BRCA promoter methylation, or additional mutations, occurs in almost half of patients with ovarian cancers resistant to PARPi therapy. Furthermore, relevant fractions of patients with other tumors, including breast, prostate, and pancreatic cancer develop resistance to PARPis [85,86,87]. Protection of stalled replication forks can occur through mutations in proteins (such as PTIP or EZH2) that lose the ability to recruit nucleases (such as MRE11 or MUS81), preventing DNA degradation [84,88]. Other mutations can affect PARP-1 binding to the replication forks and, consequently, prevent trapping by PARPis [89].

## 6. Synergy between PARPi and Radiotherapy

Although PARPis represent an unprecedented success in cancer chemotherapy, the therapeutic response ranges between 30% and 50%, and, as mentioned above, tumors develop resistance during treatment, urging additional solutions. On the other hand, tumors can also become resistant to radiotherapy, often through alterations in DNA repair pathways, with this possibility being reduced by combined chemotherapy. Noteworthily, several radioresistant tumors express PARP-1 at high levels [90,91]. In tumors exposed to IRs, PARPis could compromise DNA repair, hampering both SSB and DSB resolution and leading to DNA replication fork collapse. Although the radio-sensitizing effect is expected to be higher in BRCA1/BRCA2-mutated cancers, PARPis were shown to synergize with RT regardless of the HR proficiency [92,93]. As PARPis exert their synergic effects with RT during the S phase of the cell cycle, they could render tumor cells more susceptible to RT than nontumor slowly/nonproliferating tissue cells [94].

Hypoxia in the tumor microenvironment activates mechanisms of adaptation in tumor cells through the hypoxia-inducible factors (HIFs), which transcriptionally activate genes guiding cellular metabolism, angiogenesis, metastasis, and other processes. As IRs induce large DNA damage through the generation of ROS, hypoxia limits their effects and results in resistance to radiotherapy. In response to hypoxia, PARP-1 regulates the stability and the activity of both HIF1 and HIF2, promoting tumor cell survival. Consistently, inhibition of PARP has been shown to control tumor growth by dampening HIF activation [95,96]. Thus, PARPis could also exert a synergic therapeutic effect with RT through this mechanism.

These considerations gave rise to preclinical and clinical studies investigating the effects of PARPis as radio-sensitizers with results showing variable (limited to robust) radio-sensitizing effects [97]. Results on the toxicity of PARPis and RT combinations from clinical trials indicate that this therapeutic approach is generally safe, although hematological and gastrointestinal toxicities represent relevant adverse effects. Limitations in the available studies, including heterogeneity and reduced numbers of patients, differences in treated cancers, lack of direct comparison arms, and different RT conditions, do not yet allow reaching firm conclusions on toxicity [98].

## 7. Synergic Immunological Effects of RT and PARPi

Beyond DNA repair, several studies have shown that PARP-1 plays a relevant role in inflammation and immune responses by regulating the activation and differentiation of both innate and adaptive immune cells. Indeed, PARPis induce several immunological effects, some of which can be detrimental in cancer therapy, while others are beneficial [99,100].

Impairment of DNA repair, due to either mutations or PARPi therapy, can further sustain the damaged DNA-induced activation of the cGAS/STING pathway. Clinically approved PARPis have been shown to induce IFN-I and CCL5 expression in tumor cells trough cGAS-STING [101]. As it occurs with RT [102], the activation of this pathway by PARPis leads to CD8 T-cell recruitment at the tumor site, with the effect being more pronounced in HR-deficient triple-negative breast cancer [103]. Increased IFNγ and TNFα production by CD8 T cells and NK cells was also observed in a BRCA1-deficient ovarian cancer model upon treatment with PARPis [104]. In this model, a reduction in the frequency of MDSCs, which negatively regulate antitumor immune responses, was also induced by PARP inhibition [104]. Noteworthily, PARPis protect CD8 T cells from oxygen radical-induced apoptosis by dampening nuclear accumulation of apoptosis-inducing factor [105]. PARPi-sustained IFN-I release in the TME also promotes other relevant immune functions; it activates dendritic cells, sustains cross-presentation of tumor-derived antigens to T cells, is required for NK-cell mediated antitumor immunity, and, in synergy with TLR4 ligands, such as HMGB1, activates M1 antitumor macrophages [106,107]. Although compromised DNA repair leads to the accumulation of mutations in tumor-driving genes that can provide selective advantages in cancer cells, it also generates neoantigens that could be targeted by the immune response. Indeed, there is a favorable correlation between mutational burden and prognosis, as shown in clinics and preclinical models [108,109,110].

As discussed above, the described effects on cytokine production, cell recruitment, and mutational burden could be induced by both PARPis and RT, with synergistic effects being more likely to occur in DNA damage response-deficient tumors. In an EGFR-mutated NSCLC mouse model, niraparib increased the RT driven antitumor immunity by upregulating IFN-I production through a synergic effect on the cGAS/STING pathway. The observed reduced tumor growth and prolonged survival was associated with increased CD8 T-cell infiltration [111]. In addition, veliparib and IRs were shown to synergize in the expression of MHC-I molecules and inflammatory cytokines, as well as in calreticulin cell surface translocation, in colorectal cancer cells. Noteworthily, the effects of the PARPi and RT combination were higher in the microsatellite unstable tumor model [112].

It is worth noting that PARP inhibition also inhibits immune responses. In a pioneering study, our group showed that PARP-1 gene deletion results in higher numbers of Foxp3-expressing Treg cells in central and peripheral immunological organs [113]. Under stimulation with TGFβ, a factor that promotes tolerogenic responses in the TME [114], CD4-naïve cells from PARP-1-deficient mice differentiate to Treg cells more efficiently than wildtype CD4 cells [113]. Moreover, PARPis could upregulate PD-L1 in tumor cells by contributing to the activation of the cGAS/STING–IFN-I pathway and suppressing T-cell responses. PARPis inactivate GSK3β, a Ser/Thr protein kinase that induces phosphorylation-dependent proteasome degradation of PD-L1, resulting in PD-L1 stabilization [115,116]. Moreover, in BRCA2-deficient cells, PARPis upregulate PD-L1 through a ATM/ATR/Chk1 kinase-dependent pathway [117].

Combined PARPis and RT, therefore, have the potential to induce inflammatory signals and immunogenic cell death, as well as activate innate immune cells, consequently creating the context for the activation of the adaptive immune response toward TAA. Noteworthily, the effects are expected to be higher in genomic unstable/DNA repair compromised tumor cells, in which a wider TAA repertoire might also be generated. Effects on the expression of adhesion molecules and other alterations in the TME could contribute to immune cell recruitment and, therefore, might be useful in the treatment of tumors with a low/absent tumor immune infiltration. However, whether immune-stimulating factors induced by combined PARPis-RT prevail over suppressive elements could be sustained by synergies with further therapeutic agents (see Section 10 and Section 11).

## 8. Immune-Checkpoint Inhibitors

During last 30 years, seminal publications and subsequent studies by James Allison and Tasuku Honjo, both receiving the 2018 Nobel Prize in Physiology and Medicine [118], fostered a large wealth of studies in preclinical models and clinical trials on the use of CTLA-4 (CD152) and PD-1/PD-L1 (CD279/CD274) immune-checkpoint inhibitors (ICI) in cancer therapy.

CTLA-4, an immunoglobulin gene superfamily member discovered in activated CD8 T cells more than 30 years ago [119], is a receptor that negatively regulates cell proliferation, cytokine production, and cytotoxic functions in T cells through several mechanisms [120,121,122,123]. CTLA-4 blockade, i.e., the use of antagonist antibodies preventing CTLA-4 engagement by the natural ligands CD80 (B7.1) and CD86 (B7.2), was soon explored as a therapeutic target in tumor models by Allison [124]. As shown by several groups, CTLA-4 blockade resulted in increased effector helper and cytotoxic T-cell activity and in the reduction in immunosuppression by Treg cells [125,126]. A wealth of findings showed that it could effectively activate an immune response toward several cancer types. Conversely, in other tumor models, CTLA-4 blockade alone was not effective. In some cases, CTLA-4 effectiveness depended on the specific cell line used in the mouse model rather than on the tumor histological origin, e.g., for colon cancer models (effective with CT26 cells, not effective with MC38 cells) [127]. It soon became evident that CTLA-4 blockade as monotherapy was effective when tumors were intrinsically immunogenic, there was a lower tumor burden, and infiltrating T cells were present but not in a tolerant/exhausted status, which are all limitations suggesting to combine CTLA-4 blockade with other therapies. 

PD-1, initially discovered in activated T cells by Honjo [128], belongs to the Ig gene superfamily and is also expressed in B and NK cells, as well as in activated macrophages and dendritic cells. Stimulation of PD-1 by either PD-L1 or PD-L2 ligands negatively regulates T-cell-mediated responses including cytokine production, cell proliferation, and cytotoxic activity, although through mechanisms different from CTLA-4 [129,130]. PD-L1 and PD-L2 are expressed by antigen-presenting cells and stromal cells, and they play a relevant role in maintaining immune tolerance. PD-L1 is also expressed by some tumor cells, tumor-infiltrating leukocytes, and tumor-associated fibroblasts. PD-1 engagement on tumor-infiltrating T cells by PD-L1 inhibits their cytotoxic action toward tumor cells and leads to T-cell exhaustion, favoring tumor immune evasion [131,132]. Consistently, blockade of PD-1/PD-L1 interaction delays tumor growth, rescues CD8 T cells from exhaustion, and compromises the inhibitory activity of regulatory T cells [133,134,135]. 

CTLA-4 mainly acts in the control of T-cell activation and consequent effector T-cell generation, contributing to the maintenance of immune tolerance. Antagonistic antibodies toward CTLA-4 lower the threshold for T-cell activation and sustain the expansion of antigen-stimulated T cells, a mechanism underlying their therapeutic and toxic effects. The response can indeed be to antigens expressed on tumor and normal cells [136]. The PD-1/PD-L1 interaction plays a major role in the inhibition of tumor-infiltrating effector T cells, the killing function of which is restored by the antagonistic action of the anti-PD-1 or anti-PD-L1 antibodies. Combining CTLA-4 and PD-1 ICI enhanced the therapeutic effect compared with either therapy alone in melanoma patients [137]. 

The anti-CTLA-4 antibody ipilimumab (Yervoy) was the first approved ICI recommended for the therapy of melanoma in 2011, followed a few years later by the anti-PD-1 nivolumab for non-small-cell lung cancer (NSCLC). During the following decade, several other ICIs targeting CTLA-4 (tremelimumab in October 2022), PD1 (pembrolizumab, cemiplimab, and dostarlimab), and PD-L1 (atezolizumab, avelumab, and durvalumab) were progressively approved for clinical use as single or combined therapies.

ICI achieved considerable therapeutic success in a certain number of (advanced/metastatic) cancers including melanoma, squamous and non-squamous NSCLC, cutaneous squamous cell carcinoma, head and neck squamous cell carcinoma, Merkel cell carcinoma, and lung, gastric, and urothelial cancer [125,136,138,139,140,141,142,143,144,145,146]. Noteworthily, clinical use of ICI showed that, among the most responsive cancers, there is a subset of tumors characterized by microsatellite instability/DNA mismatch repair deficiency. These tumors display a high number of somatic mutations, leading to the expression of several neo-epitopes/neoantigens [147]. This association between clinical benefit and tumor mutational burden (TMB) was first shown with ipilimumab (anti-CTLA-4) in advanced melanoma patients [148,149]. Mismatch repair deficiency with consequently high TMB was shown to predict the response of colon cancer and later on of other solid tumors to PD-1 blockade [150].

In spite of the large therapeutic success, ICIs still fail to stably control tumor growth for a long time or to prevent recurrence in a large number of patients, with efficacy drastically varying among cancer types and within the same tumor tissue cohort [151]. The therapeutic response achieved is 10–60% in melanoma, 20–45% in NSCLC, 25% in renal cell carcinoma, and less than 20% in head and neck squamous carcinoma. In some of these patients, an initially evident primary resistance is observed, while, in others, tumors seem to acquire resistance to immunotherapy with time. For example, in about 30% of melanoma patients responding to ICI during the initial phase, their tumors become resistant during the therapy. Overall, especially for some common cancers, such as breast and prostate cancers, success is still low [152,153]. Moreover, recent studies have also shown that inhibition of a single immune checkpoint leads to the upregulation of other inhibitory receptors, likely due to a compensatory mechanism [154]. Although simultaneous blockade of more checkpoints can be successful and new immune checkpoint targets (LAG-3, TIM-3, TIGIT) are being explored [155,156], combined therapies using (more) ICIs and RT open more promising pathways.

## 9. Synergy between Radiotherapy and ICI

As discussed above, local tumor irradiation has the potential to generate an immune response against the targeted tumor. Such a response would also be expected to act on metastatic lesions that share antigenic characteristics with the original tumor, providing protection even toward not yet diagnosed secondary lesions. Conversely, irradiation of primary lesions alone does not usually elicit an effective potent antitumor immune response: local recurrences are frequent, and immune-mediated regression of distant tumors (abscopal effect) is very rare [157].

Effectiveness of ICI therapy relies on the ability of tumor cells to potentially prime an immune response, a feature depending on tumor cell intrinsic characteristics, (induced) TMB, and other TME factors. TAAs might be targeted by the immune system, provided they will be taken up and presented by APCs to T cells in an immunogenic context; T cells are primed and differentiate to effector cells, which can infiltrate tumors and possibly kill cancer cells [37].

In this context, ICIs targeting CTLA-4 can lower the threshold of activation and synergies with the RT-induced immunogenic factors (release of DAMPS, upregulation of IFN-I and MHC expression, and others described above) unleashing both CD4 and CD8 T-cell responses. PD-1/PD-L1 targeting can synergize with RT by sustaining the action of effector T cells and by reinvigorating exhausted T cells, also contrasting the effects of the RT-induced upregulation of PD-L1. As shown in experimental models (although not in all cases), in clinical trials, synergy between RT and ICI leads to immune-mediated abscopal effects, resulting in a volume reduction in distal metastasis and prevention of tumor recurrence [158].

The synergy between local RT and CTLA-4 blockade in poorly immunogenic tumors was shown in mouse models of mammary and colon carcinoma where single therapies were not effective. CTLA-4 blockade could induce an abscopal effect on metastatic lesions, when primary tumors were locally irradiated, with the effect showing a correlation with the frequency of tumor-specific IFNγ-secreting CD8 T cells [159,160]. Remarkably, the abscopal effects and the activation of tumor-specific T cells were more evident when the radiation dose was hypo-fractionated compared with a single high dose or a higher number of lower fractions [159]. The relevance of dose and dose fractionation to the TME, tumor-infiltrating leukocyte populations, cytokine production, and expression of several immunologically active factors was recently reviewed [161]. From a mechanistic point of view, the synergy between RT and CTLA-4 blockade results in the expansion of TAA-specific CD8 TILs, with the RT broadening the TCR repertoire and the anti-CTLA-4 mAb promoting activation and expansion of these selected T-cell clones [162]. Experimental models have shown that RT combined with ICI targeting PD-1/PD-L1 improved survival in mice with melanoma, breast cancer, NSCLC, and glioma [163,164,165].

How RT and immunotherapy can effectively synergize also depends on the sequencing and timing of therapies. In a syngeneic colorectal mouse model, the abscopal response was potently induced when the anti-PD-1 blocking antibody was administered after local tumor RT, resulting in distal tumor regression, expansion of functional CD8 T cells, and reduction in exhausted CD8 T cells. Conversely, when administered before RT, the anti-PD-1 antibody resulted in radio-sensitization of CD8 T cells, leading to CD8 T-cell apoptosis, and compromised the systemic immune response [166]. Another study showed that an anti-PD-L1 mAb provided a therapeutic improvement when administered in concomitance with fractionated RT but not when given sequentially [167]. At variance, CTLA-4 blockade was more effective when administered before hypo-fractionated RT, because of its depleting effect on Treg cells, rather than for its action on effector T cells. Interestingly, the same study showed that an agonistic antibody targeting OX40 was more effective when given 1 day after RT [168]. Thus, the most effective time schedule of ICI and RT administrations depends on the mechanism of action of the specific ICI used, an aspect that should be carefully considered when designing clinical trials combining ICI and RT.

The effect of synergy between RT and ipilimumab on the abscopal effect and improved survival was shown in melanoma patients [169,170,171] and later confirmed in larger studies [172,173]. After an early study showed the durable abscopal effect of RT and ipilimumab in a single patient [169], RT and CTLA-4 blockade were shown to induce systemic antitumor T-cell responses in metastatic chemo-refractory NSCLC in a larger clinical trial [174]. In NSCLC, synergy between RT and PD-L1 (nurvalumab) or PD-1 (nivolumab and pembrolizumab) ICIs was observed, with beneficial effects on progression-free survival and overall survival. In stage IV NSLC, combinations of ipilimumab and nivolumab with RT and chemotherapy were also explored [175,176,177,178,179]. Beyond melanoma and NSCLC, synergy between RT and ICI is also emerging for prostate, head and neck, and colorectal cancers, with several trials ongoing also for other cancers [38,180].

## 10. Synergy between PARPi and ICI

Therapeutic strategies combining PARPis and immunotherapy began to be explored recently. By compromising HR in tumor cells, PARPis can generate unrecoverable DNA damage, leading to increased TMB and generation of neoantigens. The generation of potentially immunogenic neoantigens correlates with better prognosis, as already mentioned above, and can synergize with ICI, improving the therapeutic response [34,108,109,110,148,181,182].

PARP inhibition affects the TME. By boosting the cGAS/STING pathway, PARPis sustain inflammation and the secretion of IFN-I and several other cytokines and chemokines, resulting in recruitment of immune cells, including tumor antigen-specific CD8 T cells. These effects could be further enhanced by ICI and are particularly relevant in those tumors otherwise cold from the immune infiltration point of view [183]. Using a BRCA1-deficient ovarian cancer mouse model, PARPi was shown to increase the therapeutic effects of CTLA-4 blockade which, as a single therapy, had limited benefit. The therapeutic effect was dependent on T-cell responses and generated a protective immunological memory. Interestingly, in this study, PARPi did not show synergic effects with PD-1/PD-L1 blockade [184]. In contrast, in another model of BRCA1-deficient ovarian cancer, PARP inhibition induced a therapeutic effect through STING activation, showing synergic effects with PD-1 blockade on antitumor T-cell response and survival [185]. A synergic reduction in tumor growth was also observed when combining PARPis and PD-L1 blockade [116]. Noteworthily, in a further study, PARPis synergized with PD-L1 blockade independently of BRCA deficiency [186]. Synergic immunological effects between PARPis and anti-PD-L1 leading to tumor growth control were also observed in a small-cell lung carcinoma mouse model [187].

Several clinical trials in phase I/II evaluated the association of PARPis and ICI (targeting CTLA-4, PD-1, or PD-L1) in triple-negative breast, ovarian, and prostate cancers. Some of these trials are still ongoing, whereas other have already published (partial) results. The combination of olaparib and tremelimumab (anti-CTLA-4) was tolerable in recurrent BRCA-associated ovarian cancer, with preliminary results showing evidence of therapeutic effect [188]. Combinations of PARPis (olaparib, pamiparib, and niraparib) and anti-PD-L1 (durvalumab) and of PARPis and anti-PD-1 (pembrolizumab and tislelizumab) were also shown to be well tolerated and associated in some cases, with a certain clinical benefit [189,190,191]. Olaparib and atezolimumab (anti-PD-L1) increased IFNγ, TNFα, and CXCL9/CXCL10 expression and tumor infiltration by lymphocytes. Although the clinical activity in recurrent ovarian cancer was modest, the increased IFNγ production was associated with improved progression-free survival [192].

In spite of the proven clinical activity, a large number of patients do not respond to the combination of PARPis and PD-L1 blockade, underlying the need for predictive biomarkers to select patients that might benefit from this combined therapy. In platinum-resistant ovarian cancer patients treated with niraparib and pembrolizumab, Färkkilä et al. identified the presence of a mutational signature (surrogate of HR deficiency) and/or the presence of IFN-primed exhausted effector CD8 T cells in the TME, to be associated with prolonged progression-free survival. Absence of both features was associated with a lack of response to niraparib plus pembrolizumab [193].

## 11. Conclusions: Combining RT, PARPis, and ICIs to Overcome Respective Limitations

As described above RT, PARPis and ICI have a certain therapeutic success when used alone, but it is their combination that can result in a better and prolonged disease control. RT and PARPis synergize in inducing DNA damage and tumor cell death. Their action results in improved therapeutic effects in preclinical models and, more recently, with studies still ongoing, in clinical settings. RT and PARPis also induce immune-stimulating factors (mainly through cGAS/STING and IFN-I), potentially generating an immunogenic microenvironment and favoring immune infiltration. However, they also activate immune-suppressive mechanisms, including the expression of PD-L1 and the recruitment of regulatory T cells and MDSCs, generating an immunosuppressive TME (Figure 2A). In fact, the induction of a systemic immune response with abscopal effects remains uncommon and/or limited. On the other hand, ICIs can lower the threshold for immune activation (mainly CTLA-4 blockade), reinvigorate exhausted T cells (mainly PD-1/PD-L1 blockade), and dampen the action of regulatory T cells, consequently sustaining systemic immune responses and the abscopal effect. Nevertheless, to be effective, they require a TME that allows priming of immune responses to tumor-associated antigens and tumor infiltration by leukocytes (Figure 2B). Combinations of immunotherapy with therapies that favor priming of immune responses, such as RT, have obtained important therapeutic success in clinical studies, with protocols including different forms of RT and ICIs having been approved for several (advanced) cancers. Furthermore, the more recent association of PARPis and ICIs showed some clinical benefits. Altogether, these results and the considerations expressed above encourage the use of combined therapies that include RT, PARPis, and ICIs.

Promising results from initial studies in experimental models have confirmed that the triple combination of RT, PARPis, and ICIs improves tumor infiltration, as well as primes and unleashes antitumor, T-cell-mediated, immune responses in mouse models. The triple combination of sub-ablative RT, olaparib, and anti-PD-1 inhibited tumor growth to a higher extent than single or two-by-two combined therapies in both microsatellite stable and unstable colon cancer [112]. The combination of RT, niraparib, and anti-PD-1 increased median survival time and reduced tumor volume in a small-cell lung carcinoma (SCLC) mouse model [194].

Several phase I–III clinical trials, aimed at exploring different combinations of radiotherapy, PARPis, and ICIs, included at least one arm with the concomitant or sequential use of these three therapeutic agents (often in addition to standard chemotherapy). The effects of PARPis together with RT and ICI, targeting CTLA-4 and/or PD-1/PD-L1 pathways, will be assessed in NSCLC, SCLC, breast, prostate, pancreatic, gastroesophageal, rectal, and head and neck carcinomas. Many of these trials are still recruiting or not yet active. Results will be available in forthcoming years (Table 1) [195].

There are still open questions regarding the identification of optimal sequencing, doses, and time intervals among the three treatments. PARPis display a radio-sensitizing action, suggesting their use along with RT. However, PARPis/RT cotreatment could also increase the risk of toxic/adverse effects, predominantly hematological or gastrointestinal, according to the targeted tissues [98]. Yet, administration of PARPis could allow reductions in RT doses, especially when associated with high-LET health tissue-sparing RT (protons and carbon ion particles), thus reducing risks. CTLA-4 blockade, by lowering the threshold for immune activation, would be relevant in the initial phases of the immune response, close to the PARPis/RT-induced “priming”. Blockade of other immune checkpoints, such as the PD-1/PD-L1 axis, might be useful after the initial “priming” to unleash the response of effector immune cells. Of note, some experimental evidence has revealed that PD-1 blockade could radio-sensitize CD8 T cells when given before RT, resulting in an unfavorable effect [166], as already discussed above. In this regard, results from the ongoing clinical trials will be informative and relevant to address toxicity and effectiveness of the three combined treatments. However, for ethical reasons, they also cannot explore many combinations exposing patients to the risk of therapeutic benefits lower than those provided by already standardized approved protocols. At variance, preclinical studies could contribute to better understand temporal sequencing and combined mechanisms of action and toxicity, helping to define clinical protocols, provided that suitable animal models will be specifically set up.

## Figures and Tables

**Table 1:** Recently approved clinical trials using combinations of RT, PARPis, and ICIs.

Title	Conditions	Therapies	Phase	Estimated Enrollment (Patients)	Status	Estimated Completion Dates	NCT Number	Last Update Posted
Testing the safety of the anticancer drugs durvalumab and olaparib during radiation therapy for locally advanced unresectable pancreatic cancer	Locally advanced pancreatic carcinoma Stage II or III pancreatic cancer Unresectable pancreatic carcinoma	Durvalumab Olaparib RT	I	18	Recruiting	Primary and final: 31 March 2024	05411094	1 December 2022
A safety study adding niraparib and dostarlimab to radiation therapy for rectal cancers	Rectal neoplasms Rectal neoplasm malignant	Niraparib Dostarlimab Short course RT	I–II	38	Recruiting	Primary: 31 December 2024 Final: 31 December 2026	04926324	26 July 2022
Niraparib + dostarlimab + RT in pancreatic cancer	Pancreatic cancer Metastatic pancreatic cancer	Niraparib Dostarlimab RT	II	25	Active, not recruiting	Primary: 19 January 2022 Final: October 2026	04409002	8 September 2022
Radiation, immunotherapy, and PARP inhibitor in triple-negative breast cancer	Breast cancer TNBC	Niraparib Dostarlimab RT	II	32	Recruiting	Primary: 1 April 2023 Final: 1 December 2029	04837209	23 December 2022
Radiotherapy and durvalumab/durvalumab combo (tremelimumab/olaparid) for small-cell lung cancer	SCLC extensive stage SCLC	Durvalumab Tremelimumab Olaparib Thoracic RT	I	25	Active, not recruiting	Primary and final: 1 June 2023	03923270	6 January 2023
A study of radiation therapy with pembrolizumab and olaparib or other radiosensitizers in women who have triple-negative or hormone-receptor positive/HER2 negative breast cancer	TNBC Metastatic breast cancer	Pembrolizumab Olaparib RT	II	34	Recruiting	Primary and final: January 2025	04683679	21 October 2022
Pembro with radiation with or without olaparib	Prostate cancer	Pembrolizumab Olaparib Androgen deprivation therapy RT	II	64	Not yet recruiting	Primary: 2 January 2025 Final: 2 January 2028	05568550	5 October 2022
Olaparib and durvalumab with carboplatin, etoposide, and/or radiation therapy for the treatment of extensive-stage small-cell lung cancer, PRIO trial	Extensive-stage SCLC Stage IV lung cancer Stage IVA lung cancer Stage IVB lung cancer	Carboplatin Durvalumab Etoposide Olaparib RT	I–II	63	Recruiting	Primary and final: 31 January 2024	04728230	9 November 2022
Study of SBRT/olaparib followed by pembrolizumab/olaparib in gastric cancers	Gastric cancer Gastroesophageal cancer	Pembrolizumab Olaparib SBRT	II	26	Recruiting	Primary: December 2025 Final: December 2028	05379972	5 January 2023
Placebo-controlled study of concurrent chemoradiation therapy with pembrolizumab followed by pembrolizumab and olaparib in newly diagnosed treatment-naïve limited-stage small-cell lung cancer (LS-SCLC) (MK 7339-013/KEYLYNK-013)	SCLC	Pembrolizumab (2 doses) Olaparib Etoposide Platinum Standard thoracic RT Prophylactic cranial irradiation	III	672	Recruiting	Primary and final: 28 October 2027	04624204	23 December 2022
Pembrolizumab plus olaparib in LA-HNSCC	Head and neck squamous cell carcinoma	Pembrolizumab Olaparib Cisplatin IMRT	II	45	Recruiting	Primary: 31 October 2024 Final: 31 October 2025	05366166	28 October 2022
Study of pembrolizumab with concurrent chemoradiation therapy, followed by pembrolizumab with or without olaparib in stage III non-small-cell lung cancer (NSCLC) (MK-7339-012/KEYLYNK-012)	Lung neoplasms NSCLC	Pembrolizumab Olaparib Etoposide Carboplatin Cisplatin Paclitaxel Pemetrexed Thoracic RT Durvalumab	III	870	Recruiting	Primary and final: 6 July 2026	04380636	30 November 2022

Listed clinical trials include at least one arm with patients undergoing treatment with RT, PARPis, and ICIs. IMRT, intensity-modulated RT; NSCLC, non-SCLC; RT, radiotherapy; SBRT, stereotactic body RT; SCLC, small-cell lung carcinoma; TNBS, triple-negative breast cancer. Source: www.clinicaltrials.gov (last accessed 8 January 2023).

## References

[1] Kocarnik J.M., Compton K., Dean F.E., Fu W., Gaw B.L., Harvey J.D., Henrikson H.J., Lu D., Pennini A., Global Burden of Disease 2019 Cancer Collaboration (2022). Cancer Incidence, Mortality, Years of Life Lost, Years Lived With Disability, and Disability-Adjusted Life Years for 29 Cancer Groups From 2010 to 2019: A Systematic Analysis for the Global Burden of Disease Study 2019. JAMA Oncol..

[2] Baumann M., Krause M., Overgaard J., Debus J., Bentzen S.M., Daartz J., Richter C., Zips D., Bortfeld T. (2016). Radiation oncology in the era of precision medicine. Nat. Rev. Cancer.

[3] Sutherland B.M., Bennett P.V., Sutherland J.C., Laval J. (2002). Clustered DNA damages induced by x rays in human cells. Radiat. Res..

[4] Goodhead D.T. (1994). Initial events in the cellular effects of ionizing radiations: Clustered damage in DNA. Int. J. Radiat. Biol..

[5] Lu Z., Zheng X., Ding C., Zou Z., Liang Y., Zhou Y., Li X. (2022). Deciphering the Biological Effects of Radiotherapy in Cancer Cells. Biomolecules.

[6] Alterio D., Gugliandolo S.G., Augugliaro M., Marvaso G., Gandini S., Bellerba F., Russell-Edu S.W., De Simone I., Cinquini M., Starzyńska A. (2021). IMRT versus 2D/3D conformal RT in oropharyngeal cancer: A review of the literature and meta-analysis. Oral Dis..

[7] Marta G.N., Silva V., de Andrade Carvalho H., de Arruda F.F., Hanna S.A., Gadia R., da Silva J.L., Correa S.F., Vita Abreu C.E., Riera R. (2014). Intensity-modulated radiation therapy for head and neck cancer: Systematic review and meta-analysis. Radiother. Oncol..

[8] Byun H.K., Han M.C., Yang K., Kim J.S., Yoo G.S., Koom W.S., Kim Y.B. (2021). Physical and Biological Characteristics of Particle Therapy for Oncologists. Cancer Res. Treat..

[9] Yuan T.Z., Zhan Z.J., Qian C.N. (2019). New frontiers in proton therapy: Applications in cancers. Cancer Commun..

[10] Zhang W., Hu W., Hu J., Gao J., Yang J., Kong L., Lu J.J. (2020). Carbon ion radiation therapy for sinonasal malignancies: Promising results from 2282 cases from the real world. Cancer Sci..

[11] Najafi M., Motevaseli E., Shirazi A., Geraily G., Rezaeyan A., Norouzi F., Rezapoor S., Abdollahi H. (2018). Mechanisms of inflammatory responses to radiation and normal tissues toxicity: Clinical implications. Int. J. Radiat. Biol..

[12] Rodríguez-Ruiz M.E., Vanpouille-Box C., Melero I., Formenti S.C., Demaria S. (2018). Immunological Mechanisms Responsible for Radiation-Induced Abscopal Effect. Trends Immunol..

[13] Deng L., Liang H., Xu M., Yang X., Burnette B., Arina A., Li X.-D., Mauceri H., Beckett M., Darga T. (2014). STING-Dependent Cytosolic DNA Sensing Promotes Radiation-Induced Type I Interferon-Dependent Antitumor Immunity in Immunogenic Tumors. Immunity.

[14] Yamazaki T., Galluzzi L. (2020). Mitochondrial control of innate immune signaling by irradiated cancer cells. OncoImmunology.

[15] Burnette B.C., Liang H., Lee Y., Chlewicki L., Khodarev N.N., Weichselbaum R.R., Fu Y.-X., Auh S.L. (2011). The Efficacy of Radiotherapy Relies upon Induction of Type I Interferon–Dependent Innate and Adaptive Immunity. Cancer Res..

[16] Du J., Kageyama S.I., Hirata H., Motegi A., Nakamura M., Hirano Y., Okumura M., Yamashita R., Tsuchihara K., Hojo H. (2021). Comparative analysis of the immune responses in cancer cells irradiated with X-ray, proton and carbon-ion beams. Biochem. Biophys. Res. Commun..

[17] Cytlak U.M., Dyer D.P., Honeychurch J., Williams K.J., Travis M.A., Illidge T.M. (2022). Immunomodulation by radiotherapy in tumour control and normal tissue toxicity. Nat. Rev. Immunol..

[18] Connolly K.A., Belt B.A., Figueroa N.M., Murthy A., Patel A., Kim M., Lord E.M., Linehan D.C., Gerber S.A. (2016). Increasing the efficacy of radiotherapy by modulating the CCR2/CCR5 chemokine axes. Oncotarget.

[19] Barker H.E., Paget J.T., Khan A.A., Harrington K.J. (2015). The tumour microenvironment after radiotherapy: Mechanisms of resistance and recurrence. Nat. Rev. Cancer.

[20] Li H., Chen X., Zeng W., Zhou W., Zhou Q., Wang Z., Jiang W., Xie B., Sun L.Q. (2020). Radiation-Enhanced Expression of CCL22 in Nasopharyngeal Carcinoma is Associated With CCR4(+) CD8 T Cell Recruitment. Int. J. Radiat. Oncol. Biol. Phys..

[21] Matsumura S., Wang B., Kawashima N., Braunstein S., Badura M., Cameron T.O., Babb J.S., Schneider R.J., Formenti S.C., Dustin M.L. (2008). Radiation-induced CXCL16 release by breast cancer cells attracts effector T cells. J. Immunol..

[22] Cheng C.C., Chang Y.F., Ho A.S., Sie Z.L., Chang J.S., Peng C.L., Chang C.C. (2021). Irradiation Mediates IFNα and CXCL9 Expression in Non-Small Cell Lung Cancer to Stimulate CD8(+) T Cells Activity and Migration toward Tumors. Biomedicines.

[23] Hallahan D., Kuchibhotla J., Wyble C. (1996). Cell adhesion molecules mediate radiation-induced leukocyte adhesion to the vascular endothelium. Cancer Res..

[24] Krysko D.V., Garg A.D., Kaczmarek A., Krysko O., Agostinis P., Vandenabeele P. (2012). Immunogenic cell death and DAMPs in cancer therapy. Nat. Rev. Cancer.

[25] Gong T., Liu L., Jiang W., Zhou R. (2019). DAMP-sensing receptors in sterile inflammation and inflammatory diseases. Nat. Rev. Immunol..

[26] Apetoh L., Ghiringhelli F., Tesniere A., Obeid M., Ortiz C., Criollo A., Mignot G., Maiuri M.C., Ullrich E., Saulnier P. (2007). Toll-like receptor 4-dependent contribution of the immune system to anticancer chemotherapy and radiotherapy. Nat. Med..

[27] Ghiringhelli F., Apetoh L., Tesniere A., Aymeric L., Ma Y., Ortiz C., Vermaelen K., Panaretakis T., Mignot G., Ullrich E. (2009). Activation of the NLRP3 inflammasome in dendritic cells induces IL-1beta-dependent adaptive immunity against tumors. Nat. Med..

[28] Gold L.I., Eggleton P., Sweetwyne M.T., Van Duyn L.B., Greives M.R., Naylor S.M., Michalak M., Murphy-Ullrich J.E. (2010). Calreticulin: Non-endoplasmic reticulum functions in physiology and disease. FASEB J..

[29] Gardai S.J., McPhillips K.A., Frasch S.C., Janssen W.J., Starefeldt A., Murphy-Ullrich J.E., Bratton D.L., Oldenborg P.A., Michalak M., Henson P.M. (2005). Cell-surface calreticulin initiates clearance of viable or apoptotic cells through trans-activation of LRP on the phagocyte. Cell.

[30] Gameiro S.R., Malamas A.S., Bernstein M.B., Tsang K.Y., Vassantachart A., Sahoo N., Tailor R., Pidikiti R., Guha C.P., Hahn S.M. (2016). Tumor Cells Surviving Exposure to Proton or Photon Radiation Share a Common Immunogenic Modulation Signature, Rendering Them More Sensitive to T Cell–Mediated Killing. Int. J. Radiat. Oncol. Biol..

[31] Böttcher J.P., Bonavita E., Chakravarty P., Blees H., Cabeza-Cabrerizo M., Sammicheli S., Rogers N.C., Sahai E., Zelenay S., Reis e Sousa C. (2018). NK Cells Stimulate Recruitment of cDC1 into the Tumor Microenvironment Promoting Cancer Immune Control. Cell.

[32] Krombach J., Hennel R., Brix N., Orth M., Schoetz U., Ernst A., Schuster J., Zuchtriegel G., Reichel C.A., Bierschenk S. (2019). Priming anti-tumor immunity by radiotherapy: Dying tumor cell-derived DAMPs trigger endothelial cell activation and recruitment of myeloid cells. Oncoimmunology.

[33] Reits E.A., Hodge J.W., Herberts C.A., Groothuis T.A., Chakraborty M.K., Wansley E., Camphausen K., Luiten R.M., de Ru A.H., Neijssen J. (2006). Radiation modulates the peptide repertoire, enhances MHC class I expression, and induces successful antitumor immunotherapy. J. Exp. Med..

[34] Schumacher T.N., Schreiber R.D. (2015). Neoantigens in cancer immunotherapy. Science.

[35] Obeid M., Tesniere A., Ghiringhelli F., Fimia G.M., Apetoh L., Perfettini J.L., Castedo M., Mignot G., Panaretakis T., Casares N. (2007). Calreticulin exposure dictates the immunogenicity of cancer cell death. Nat. Med..

[36] Lee Y., Auh S.L., Wang Y., Burnette B., Wang Y., Meng Y., Beckett M., Sharma R., Chin R., Tu T. (2009). Therapeutic effects of ablative radiation on local tumor require CD8+ T cells: Changing strategies for cancer treatment. Blood.

[37] Chen D.S., Mellman I. (2013). Oncology meets immunology: The cancer-immunity cycle. Immunity.

[38] Procureur A., Simonaggio A., Bibault J.E., Oudard S., Vano Y.A. (2021). Enhance the Immune Checkpoint Inhibitors Efficacy with Radiotherapy Induced Immunogenic Cell Death: A Comprehensive Review and Latest Developments. Cancers.

[39] Mauch P., Constine L., Greenberger J., Knospe W., Sullivan J., Liesveld J.L., Deeg H.J. (1995). Hematopoietic stem cell compartment: Acute and late effects of radiation therapy and chemotherapy. Int. J. Radiat. Oncol. Biol. Phys..

[40] Frasca D., Guidi F., Arbitrio M., Pioli C., Poccia F., Cicconi R., Doria G. (2000). Hematopoietic reconstitution after lethal irradiation and bone marrow transplantation: Effects of different hematopoietic cytokines on the recovery of thymus, spleen and blood cells. Bone Marrow Transplant..

[41] Frasca D., Pioli C., Guidi F., Pucci S., Arbitrio M., Leter G., Doria G. (1996). IL-11 synergizes with IL-3 in promoting the recovery of the immune system after irradiation. Int. Immunol..

[42] Heylmann D., Rödel F., Kindler T., Kaina B. (2014). Radiation sensitivity of human and murine peripheral blood lymphocytes, stem and progenitor cells. Biochim. Biophys. Acta.

[43] Berte N., Eich M., Heylmann D., Koks C., Van Gool S.W., Kaina B. (2021). Impaired DNA repair in mouse monocytes compared to macrophages and precursors. DNA Repair.

[44] Leblond M.M., Pérès E.A., Helaine C., Gérault A.N., Moulin D., Anfray C., Divoux D., Petit E., Bernaudin M., Valable S. (2017). M2 macrophages are more resistant than M1 macrophages following radiation therapy in the context of glioblastoma. Oncotarget.

[45] Groves A.M., Johnston C.J., Misra R.S., Williams J.P., Finkelstein J.N. (2016). Effects of IL-4 on pulmonary fibrosis and the accumulation and phenotype of macrophage subpopulations following thoracic irradiation. Int. J. Radiat. Biol..

[46] Arina A., Beckett M., Fernandez C., Zheng W., Pitroda S., Chmura S.J., Luke J.J., Forde M., Hou Y., Burnette B. (2019). Tumor-reprogrammed resident T cells resist radiation to control tumors. Nat. Commun..

[47] Qinfeng S., Depu W., Xiaofeng Y., Shah W., Hongwei C., Yili W. (2013). In situ observation of the effects of local irradiation on cytotoxic and regulatory T lymphocytes in cervical cancer tissue. Radiat. Res..

[48] Qu Y., Jin S., Zhang A., Zhang B., Shi X., Wang J., Zhao Y. (2010). Gamma-ray resistance of regulatory CD4+CD25+Foxp3+ T cells in mice. Radiat. Res..

[49] Kachikwu E.L., Iwamoto K.S., Liao Y.P., DeMarco J.J., Agazaryan N., Economou J.S., McBride W.H., Schaue D. (2011). Radiation enhances regulatory T cell representation. Int. J. Radiat. Oncol. Biol. Phys..

[50] Marciscano A.E., Ghasemzadeh A., Nirschl T.R., Theodros D., Kochel C.M., Francica B.J., Muroyama Y., Anders R.A., Sharabi A.B., Velarde E. (2018). Elective Nodal Irradiation Attenuates the Combinatorial Efficacy of Stereotactic Radiation Therapy and Immunotherapy. Clin. Cancer Res..

[51] Yovino S., Kleinberg L., Grossman S.A., Narayanan M., Ford E. (2013). The Etiology of Treatment-related Lymphopenia in Patients with Malignant Gliomas: Modeling Radiation Dose to Circulating Lymphocytes Explains Clinical Observations and Suggests Methods of Modifying the Impact of Radiation on Immune Cells. Cancer Investig..

[52] Zhai D., An D., Wan C., Yang K. (2022). Radiotherapy: Brightness and darkness in the era of immunotherapy. Transl. Oncol..

[53] Derer A., Spiljar M., Bäumler M., Hecht M., Fietkau R., Frey B., Gaipl U.S. (2016). Chemoradiation Increases PD-L1 Expression in Certain Melanoma and Glioblastoma Cells. Front. Immunol..

[54] Gao Y., Li Y., Lin Z., Zeng Y., Huang Z., Han L., Zhong Y., Gong Y., Wu Q., Xie C. (2022). Ataxia telangiectasia mutated kinase inhibition promotes irradiation-induced PD-L1 expression in tumour-associated macrophages through IFN-I/JAK signalling pathway. Immunology.

[55] Mondini M., Loyher P.-L., Hamon P., Gerbé de Thoré M., Laviron M., Berthelot K., Clémenson C., Salomon B.L., Combadière C., Deutsch E. (2019). CCR2-Dependent Recruitment of Tregs and Monocytes Following Radiotherapy Is Associated with TNFα-Mediated Resistance. Cancer Immunol. Res..

[56] Liang H., Deng L., Hou Y., Meng X., Huang X., Rao E., Zheng W., Mauceri H., Mack M., Xu M. (2017). Host STING-dependent MDSC mobilization drives extrinsic radiation resistance. Nat. Commun..

[57] Jobling M.F., Mott J.D., Finnegan M.T., Jurukovski V., Erickson A.C., Walian P.J., Taylor S.E., Ledbetter S., Lawrence C.M., Rifkin D.B. (2006). Isoform-specific activation of latent transforming growth factor beta (LTGF-beta) by reactive oxygen species. Radiat. Res..

[58] Chiang C.S., Fu S.Y., Wang S.C., Yu C.F., Chen F.H., Lin C.M., Hong J.H. (2012). Irradiation promotes an m2 macrophage phenotype in tumor hypoxia. Front. Oncol..

[59] Farhood B., Khodamoradi E., Hoseini-Ghahfarokhi M., Motevaseli E., Mirtavoos-Mahyari H., Eleojo Musa A., Najafi M. (2020). TGF-β in radiotherapy: Mechanisms of tumor resistance and normal tissues injury. Pharmacol. Res..

[60] Costa A., Kieffer Y., Scholer-Dahirel A., Pelon F., Bourachot B., Cardon M., Sirven P., Magagna I., Fuhrmann L., Bernard C. (2018). Fibroblast Heterogeneity and Immunosuppressive Environment in Human Breast Cancer. Cancer Cell.

[61] Liao Y., Liu S., Fu S., Wu J. (2020). HMGB1 in Radiotherapy: A Two Headed Signal Regulating Tumor Radiosensitivity and Immunity. OncoTargets Ther..

[62] Shrivastava S., Mansure J.J., Almajed W., Cury F., Ferbeyre G., Popovic M., Seuntjens J., Kassouf W. (2016). The Role of HMGB1 in Radioresistance of Bladder Cancer. Mol. Cancer Ther..

[63] He S., Cheng J., Sun L., Wang Y., Wang C., Liu X., Zhang Z., Zhao M., Luo Y., Tian L. (2018). HMGB1 released by irradiated tumor cells promotes living tumor cell proliferation via paracrine effect. Cell Death Dis..

[64] Condamine T., Gabrilovich D.I. (2011). Molecular mechanisms regulating myeloid-derived suppressor cell differentiation and function. Trends Immunol..

[65] Beavis P.A., Stagg J., Darcy P.K., Smyth M.J. (2012). CD73: A potent suppressor of antitumor immune responses. Trends Immunol..

[66] Ohta A., Gorelik E., Prasad S.J., Ronchese F., Lukashev D., Wong M.K., Huang X., Caldwell S., Liu K., Smith P. (2006). A2A adenosine receptor protects tumors from antitumor T cells. Proc. Natl. Acad. Sci. USA.

[67] Stagg J., Beavis P.A., Divisekera U., Liu M.C., Möller A., Darcy P.K., Smyth M.J. (2012). CD73-deficient mice are resistant to carcinogenesis. Cancer Res..

[68] Michaud M., Sukkurwala A.Q., Martins I., Shen S., Zitvogel L., Kroemer G. (2012). Subversion of the chemotherapy-induced anticancer immune response by the ecto-ATPase CD39. Oncoimmunology.

[69] Krishnakumar R., Kraus W.L. (2010). The PARP side of the nucleus: Molecular actions, physiological outcomes, and clinical targets. Mol. Cell.

[70] El-Khamisy S.F., Masutani M., Suzuki H., Caldecott K.W. (2003). A requirement for PARP-1 for the assembly or stability of XRCC1 nuclear foci at sites of oxidative DNA damage. Nucleic Acids Res..

[71] Mladenov E., Iliakis G. (2011). Induction and repair of DNA double strand breaks: The increasing spectrum of non-homologous end joining pathways. Mutat. Res. Fundam. Mol. Mech. Mutagen..

[72] Langelier M.F., Planck J.L., Roy S., Pascal J.M. (2012). Structural basis for DNA damage-dependent poly(ADP-ribosyl)ation by human PARP-1. Science.

[73] Wang M., Wu W., Wu W., Rosidi B., Zhang L., Wang H., Iliakis G. (2006). PARP-1 and Ku compete for repair of DNA double strand breaks by distinct NHEJ pathways. Nucleic Acids Res..

[74] Ahmed E.A., Alzahrani A.M., Scherthan H. (2021). Parp1-Dependent DNA Double-Strand Break Repair in Irradiated Late Pachytene Spermatocytes. DNA Cell Biol..

[75] Robson M., Im S.A., Senkus E., Xu B., Domchek S.M., Masuda N., Delaloge S., Li W., Tung N., Armstrong A. (2017). Olaparib for Metastatic Breast Cancer in Patients with a Germline BRCA Mutation. N. Engl. J. Med..

[76] Litton J.K., Rugo H.S., Ettl J., Hurvitz S.A., Gonçalves A., Lee K.H., Fehrenbacher L., Yerushalmi R., Mina L.A., Martin M. (2018). Talazoparib in Patients with Advanced Breast Cancer and a Germline BRCA Mutation. N. Engl. J. Med..

[77] Kaelin W.G. (2005). The concept of synthetic lethality in the context of anticancer therapy. Nat. Rev. Cancer.

[78] Lord C.J., Ashworth A. (2017). PARP inhibitors: Synthetic lethality in the clinic. Science.

[79] Bryant H.E., Schultz N., Thomas H.D., Parker K.M., Flower D., Lopez E., Kyle S., Meuth M., Curtin N.J., Helleday T. (2005). Specific killing of BRCA2-deficient tumours with inhibitors of poly(ADP-ribose) polymerase. Nature.

[80] Farmer H., McCabe N., Lord C.J., Tutt A.N., Johnson D.A., Richardson T.B., Santarosa M., Dillon K.J., Hickson I., Knights C. (2005). Targeting the DNA repair defect in BRCA mutant cells as a therapeutic strategy. Nature.

[81] Pilié P.G., Gay C.M., Byers L.A., O’Connor M.J., Yap T.A. (2019). PARP Inhibitors: Extending Benefit Beyond BRCA-Mutant Cancers. Clin. Cancer Res..

[82] Zandarashvili L., Langelier M.F., Velagapudi U.K., Hancock M.A., Steffen J.D., Billur R., Hannan Z.M., Wicks A.J., Krastev D.B., Pettitt S.J. (2020). Structural basis for allosteric PARP-1 retention on DNA breaks. Science.

[83] Murai J., Huang S.Y., Renaud A., Zhang Y., Ji J., Takeda S., Morris J., Teicher B., Doroshow J.H., Pommier Y. (2014). Stereospecific PARP trapping by BMN 673 and comparison with olaparib and rucaparib. Mol. Cancer Ther..

[84] D’Andrea A.D. (2018). Mechanisms of PARP inhibitor sensitivity and resistance. DNA Repair.

[85] Christie E.L., Fereday S., Doig K., Pattnaik S., Dawson S.J., Bowtell D.D.L. (2017). Reversion of BRCA1/2 Germline Mutations Detected in Circulating Tumor DNA From Patients With High-Grade Serous Ovarian Cancer. J. Clin. Oncol..

[86] Sakai W., Swisher E.M., Karlan B.Y., Agarwal M.K., Higgins J., Friedman C., Villegas E., Jacquemont C., Farrugia D.J., Couch F.J. (2008). Secondary mutations as a mechanism of cisplatin resistance in BRCA2-mutated cancers. Nature.

[87] Park P.H., Yamamoto T.M., Li H., Alcivar A.L., Xia B., Wang Y., Bernhardy A.J., Turner K.M., Kossenkov A.V., Watson Z.L. (2020). Amplification of the Mutation-Carrying BRCA2 Allele Promotes RAD51 Loading and PARP Inhibitor Resistance in the Absence of Reversion Mutations. Mol. Cancer Ther..

[88] Ray Chaudhuri A., Callen E., Ding X., Gogola E., Duarte A.A., Lee J.E., Wong N., Lafarga V., Calvo J.A., Panzarino N.J. (2016). Replication fork stability confers chemoresistance in BRCA-deficient cells. Nature.

[89] Pettitt S.J., Krastev D.B., Brandsma I., Dréan A., Song F., Aleksandrov R., Harrell M.I., Menon M., Brough R., Campbell J. (2018). Genome-wide and high-density CRISPR-Cas9 screens identify point mutations in PARP1 causing PARP inhibitor resistance. Nat. Commun..

[90] Kupczyk P., Simiczyjew A., Marczuk J., Dratkiewicz E., Beberok A., Rok J., Pieniazek M., Biecek P., Nevozhay D., Slowikowski B. (2021). PARP1 as a Marker of an Aggressive Clinical Phenotype in Cutaneous Melanoma—A Clinical and an In Vitro Study. Cells.

[91] Raleigh D., Ahmed K.M., Zhang H., Ziaee S., Park C.C. (2016). PARP-1 modulates β1-integrin/NF-κB-mediated radioresistance in human breast cancer. J. Cancer Ther. Res..

[92] Cerrato A., Morra F., Celetti A. (2016). Use of poly ADP-ribose polymerase [PARP] inhibitors in cancer cells bearing DDR defects: The rationale for their inclusion in the clinic. J. Exp. Clin. Cancer Res..

[93] Zhao W., Hu H., Mo Q., Guan Y., Li Y., Du Y., Li L. (2019). Function and mechanism of combined PARP-1 and BRCA genes in regulating the radiosensitivity of breast cancer cells. Int. J. Clin. Exp. Pathol..

[94] Dungey F.A., Löser D.A., Chalmers A.J. (2008). Replication-dependent radiosensitization of human glioma cells by inhibition of poly(ADP-Ribose) polymerase: Mechanisms and therapeutic potential. Int. J. Radiat. Oncol. Biol. Phys..

[95] Elser M., Borsig L., Hassa P.O., Erener S., Messner S., Valovka T., Keller S., Gassmann M., Hottiger M.O. (2008). Poly(ADP-Ribose) Polymerase 1 Promotes Tumor Cell Survival by Coactivating Hypoxia-Inducible Factor-1–Dependent Gene Expression. Mol. Cancer Res..

[96] Gonzalez-Flores A., Aguilar-Quesada R., Siles E., Pozo S., Rodríguez-Lara M.I., López-Jiménez L., López-Rodríguez M., Peralta-Leal A., Villar D., Martín-Oliva D. (2014). Interaction between PARP-1 and HIF-2α in the hypoxic response. Oncogene.

[97] Lesueur P., Chevalier F., Austry J.B., Waissi W., Burckel H., Noël G., Habrand J.L., Saintigny Y., Joly F. (2017). Poly-(ADP-ribose)-polymerase inhibitors as radiosensitizers: A systematic review of pre-clinical and clinical human studies. Oncotarget.

[98] Barcellini A., Loap P., Murata K., Villa R., Kirova Y., Okonogi N., Orlandi E. (2021). PARP Inhibitors in Combination with Radiotherapy: To Do or Not to Do?. Cancers.

[99] Rosado M.M., Bennici E., Novelli F., Pioli C. (2013). Beyond DNA repair, the immunological role of PARP-1 and its siblings. Immunology.

[100] Rosado M.M., Pioli C. (2021). ADP-ribosylation in evasion, promotion and exacerbation of immune responses. Immunology.

[101] Chabanon R.M., Muirhead G., Krastev D.B., Adam J., Morel D., Garrido M., Lamb A., Hénon C., Dorvault N., Rouanne M. (2019). PARP inhibition enhances tumor cell-intrinsic immunity in ERCC1-deficient non-small cell lung cancer. J. Clin. Investig..

[102] Lim J.Y.H., Gerber S.A., Murphy S.P., Lord E.M. (2014). Type I interferons induced by radiation therapy mediate recruitment and effector function of CD8+ T cells. Cancer Immunol. Immunother..

[103] Pantelidou C., Sonzogni O., De Oliveria Taveira M., Mehta A.K., Kothari A., Wang D., Visal T., Li M.K., Pinto J., Castrillon J.A. (2019). PARP Inhibitor Efficacy Depends on CD8(+) T-cell Recruitment via Intratumoral STING Pathway Activation in BRCA-Deficient Models of Triple-Negative Breast Cancer. Cancer Discov..

[104] Huang J., Wang L., Cong Z., Amoozgar Z., Kiner E., Xing D., Orsulic S., Matulonis U., Goldberg M.S. (2015). The PARP1 inhibitor BMN 673 exhibits immunoregulatory effects in a Brca1−/− murine model of ovarian cancer. Biochem. Biophys. Res. Commun..

[105] Thorén F.B., Romero A.I., Hellstrand K. (2006). Oxygen Radicals Induce Poly(ADP-Ribose) Polymerase-Dependent Cell Death in Cytotoxic Lymphocytes1. J. Immunol..

[106] Karimi K., Karimi Y., Chan J., Boudreau J.E., Basset J., Chew M.V., Reid S., Bramson J.L., Wan Y., Ashkar A.A. (2015). Type I IFN signaling on dendritic cells is required for NK cell-mediated anti-tumor immunity. Innate Immun..

[107] Müller E., Speth M., Christopoulos P.F., Lunde A., Avdagic A., Øynebråten I., Corthay A. (2018). Both Type I and Type II Interferons Can Activate Antitumor M1 Macrophages When Combined With TLR Stimulation. Front. Immunol..

[108] Yarchoan M., Johnson B.A., Lutz E.R., Laheru D.A., Jaffee E.M. (2017). Targeting neoantigens to augment antitumour immunity. Nat. Rev. Cancer.

[109] Germano G., Lamba S., Rospo G., Barault L., Magrì A., Maione F., Russo M., Crisafulli G., Bartolini A., Lerda G. (2017). Inactivation of DNA repair triggers neoantigen generation and impairs tumour growth. Nature.

[110] Marcus L., Lemery S.J., Keegan P., Pazdur R. (2019). FDA Approval Summary: Pembrolizumab for the Treatment of Microsatellite Instability-High Solid Tumors. Clin. Cancer Res..

[111] Zhang N., Gao Y., Zeng Z., Luo Y., Jiang X., Zhang J., Li J., Zhang J., Gong Y., Xie C. (2021). PARP inhibitor niraparib as a radiosensitizer promotes antitumor immunity of radiotherapy in EGFR-mutated non-small cell lung cancer. Clin. Transl. Oncol..

[112] Seyedin S.N., Hasibuzzaman M.M., Pham V., Petronek M.S., Callaghan C., Kalen A.L., Mapuskar K.A., Mott S.L., Spitz D.R., Allen B.G. (2020). Combination Therapy with Radiation and PARP Inhibition Enhances Responsiveness to Anti-PD-1 Therapy in Colorectal Tumor Models. Int. J. Radiat. Oncol. Biol. Phys..

[113] Nasta F., Laudisi F., Sambucci M., Rosado M.M., Pioli C. (2010). Increased Foxp3+ regulatory T cells in poly(ADP-Ribose) polymerase-1 deficiency. J. Immunol..

[114] Angioni R., Sánchez-Rodríguez R., Viola A., Molon B. (2021). TGF-β in Cancer: Metabolic Driver of the Tolerogenic Crosstalk in the Tumor Microenvironment. Cancers.

[115] Li C.-W., Lim S.-O., Xia W., Lee H.-H., Chan L.-C., Kuo C.-W., Khoo K.-H., Chang S.-S., Cha J.-H., Kim T. (2016). Glycosylation and stabilization of programmed death ligand-1 suppresses T-cell activity. Nat. Commun..

[116] Jiao S., Xia W., Yamaguchi H., Wei Y., Chen M.K., Hsu J.M., Hsu J.L., Yu W.H., Du Y., Lee H.H. (2017). PARP Inhibitor Upregulates PD-L1 Expression and Enhances Cancer-Associated Immunosuppression. Clin. Cancer Res..

[117] Sato H., Niimi A., Yasuhara T., Permata T.B.M., Hagiwara Y., Isono M., Nuryadi E., Sekine R., Oike T., Kakoti S. (2017). DNA double-strand break repair pathway regulates PD-L1 expression in cancer cells. Nat. Commun..

[118] Wolchok J. (2018). Putting the Immunologic Brakes on Cancer. Cell.

[119] Brunet J.F., Denizot F., Luciani M.F., Roux-Dosseto M., Suzan M., Mattei M.G., Golstein P. (1987). A new member of the immunoglobulin superfamily--CTLA-4. Nature.

[120] Krummel M.F., Allison J.P. (1995). CD28 and CTLA-4 have opposing effects on the response of T cells to stimulation. J. Exp. Med..

[121] Pioli C., Gatta L., Frasca D., Doria G. (1999). Cytotoxic T lymphocyte antigen 4 (CTLA-4) inhibits CD28-induced IkappaBalpha degradation and RelA activation. Eur. J. Immunol..

[122] Walunas T.L., Lenschow D.J., Bakker C.Y., Linsley P.S., Freeman G.J., Green J.M., Thompson C.B., Bluestone J.A. (1994). CTLA-4 can function as a negative regulator of T cell activation. Immunity.

[123] Gatta L., Calviello G., Di Nicuolo F., Pace L., Ubaldi V., Doria G., Pioli C. (2002). Cytotoxic T lymphocyte-associated antigen-4 inhibits integrin-mediated stimulation. Immunology.

[124] Leach D.R., Krummel M.F., Allison J.P. (1996). Enhancement of antitumor immunity by CTLA-4 blockade. Science.

[125] Seidel J.A., Otsuka A., Kabashima K. (2018). Anti-PD-1 and Anti-CTLA-4 Therapies in Cancer: Mechanisms of Action, Efficacy, and Limitations. Front. Oncol..

[126] Peggs K.S., Quezada S.A., Chambers C.A., Korman A.J., Allison J.P. (2009). Blockade of CTLA-4 on both effector and regulatory T cell compartments contributes to the antitumor activity of anti-CTLA-4 antibodies. J. Exp. Med..

[127] Grosso J.F., Jure-Kunkel M.N. (2013). CTLA-4 blockade in tumor models: An overview of preclinical and translational research. Cancer Immun..

[128] Ishida Y., Agata Y., Shibahara K., Honjo T. (1992). Induced expression of PD-1, a novel member of the immunoglobulin gene superfamily, upon programmed cell death. EMBO J..

[129] Freeman G.J., Long A.J., Iwai Y., Bourque K., Chernova T., Nishimura H., Fitz L.J., Malenkovich N., Okazaki T., Byrne M.C. (2000). Engagement of the PD-1 immunoinhibitory receptor by a novel B7 family member leads to negative regulation of lymphocyte activation. J. Exp. Med..

[130] Latchman Y., Wood C.R., Chernova T., Chaudhary D., Borde M., Chernova I., Iwai Y., Long A.J., Brown J.A., Nunes R. (2001). PD-L2 is a second ligand for PD-1 and inhibits T cell activation. Nat. Immunol..

[131] Iwai Y., Ishida M., Tanaka Y., Okazaki T., Honjo T., Minato N. (2002). Involvement of PD-L1 on tumor cells in the escape from host immune system and tumor immunotherapy by PD-L1 blockade. Proc. Natl. Acad. Sci. USA.

[132] Barber D.L., Wherry E.J., Masopust D., Zhu B., Allison J.P., Sharpe A.H., Freeman G.J., Ahmed R. (2006). Restoring function in exhausted CD8 T cells during chronic viral infection. Nature.

[133] Hirano F., Kaneko K., Tamura H., Dong H., Wang S., Ichikawa M., Rietz C., Flies D.B., Lau J.S., Zhu G. (2005). Blockade of B7-H1 and PD-1 by monoclonal antibodies potentiates cancer therapeutic immunity. Cancer Res..

[134] Miller B.C., Sen D.R., Al Abosy R., Bi K., Virkud Y.V., LaFleur M.W., Yates K.B., Lako A., Felt K., Naik G.S. (2019). Subsets of exhausted CD8+ T cells differentially mediate tumor control and respond to checkpoint blockade. Nat. Immunol..

[135] Kim M.J., Kim K., Park H.J., Kim G.-R., Hong K.H., Oh J.H., Son J., Park D.J., Kim D., Choi J.-M. (2023). Deletion of PD-1 destabilizes the lineage identity and metabolic fitness of tumor-infiltrating regulatory T cells. Nat. Immunol..

[136] Hodi F.S., O’Day S.J., McDermott D.F., Weber R.W., Sosman J.A., Haanen J.B., Gonzalez R., Robert C., Schadendorf D., Hassel J.C. (2010). Improved survival with ipilimumab in patients with metastatic melanoma. N. Engl. J. Med..

[137] Wolchok J.D., Kluger H., Callahan M.K., Postow M.A., Rizvi N.A., Lesokhin A.M., Segal N.H., Ariyan C.E., Gordon R.A., Reed K. (2013). Nivolumab plus ipilimumab in advanced melanoma. N. Engl. J. Med..

[138] Postow M.A., Callahan M.K., Wolchok J.D. (2015). Immune Checkpoint Blockade in Cancer Therapy. J. Clin. Oncol..

[139] Huang Q., Zheng Y., Gao Z., Yuan L., Sun Y., Chen H. (2021). Comparative Efficacy and Safety of PD-1/PD-L1 Inhibitors for Patients with Solid Tumors: A Systematic Review and Bayesian Network Meta-analysis. J. Cancer.

[140] Vuky J., Balar A.V., Castellano D., O’Donnell P.H., Grivas P., Bellmunt J., Powles T., Bajorin D., Hahn N.M., Savage M.J. (2020). Long-Term Outcomes in KEYNOTE-052: Phase II Study Investigating First-Line Pembrolizumab in Cisplatin-Ineligible Patients With Locally Advanced or Metastatic Urothelial Cancer. J. Clin. Oncol..

[141] Shiravand Y., Khodadadi F., Kashani S.M.A., Hosseini-Fard S.R., Hosseini S., Sadeghirad H., Ladwa R., O’Byrne K., Kulasinghe A. (2022). Immune Checkpoint Inhibitors in Cancer Therapy. Curr. Oncol..

[142] Mullard A. (2022). Second CTLA4-targeted checkpoint inhibitor secures FDA approval. Nat. Rev. Drug Discov..

[143] Robert C. (2020). A decade of immune-checkpoint inhibitors in cancer therapy. Nat. Commun..

[144] Brahmer J.R., Tykodi S.S., Chow L.Q.M., Hwu W.-J., Topalian S.L., Hwu P., Drake C.G., Camacho L.H., Kauh J., Odunsi K. (2012). Safety and Activity of Anti–PD-L1 Antibody in Patients with Advanced Cancer. N. Engl. J. Med..

[145] Forde P.M., Chaft J.E., Smith K.N., Anagnostou V., Cottrell T.R., Hellmann M.D., Zahurak M., Yang S.C., Jones D.R., Broderick S. (2018). Neoadjuvant PD-1 Blockade in Resectable Lung Cancer. N. Engl. J. Med..

[146] Schmid P., Adams S., Rugo H.S., Schneeweiss A., Barrios C.H., Iwata H., Diéras V., Hegg R., Im S.-A., Shaw Wright G. (2018). Atezolizumab and Nab-Paclitaxel in Advanced Triple-Negative Breast Cancer. N. Engl. J. Med..

[147] Nebot-Bral L., Brandao D., Verlingue L., Rouleau E., Caron O., Despras E., El-Dakdouki Y., Champiat S., Aoufouchi S., Leary A. (2017). Hypermutated tumours in the era of immunotherapy: The paradigm of personalised medicine. Eur. J. Cancer.

[148] Van Allen E.M., Miao D., Schilling B., Shukla S.A., Blank C., Zimmer L., Sucker A., Hillen U., Geukes Foppen M.H., Goldinger S.M. (2015). Genomic correlates of response to CTLA-4 blockade in metastatic melanoma. Science.

[149] Snyder A., Makarov V., Merghoub T., Yuan J., Zaretsky J.M., Desrichard A., Walsh L.A., Postow M.A., Wong P., Ho T.S. (2014). Genetic basis for clinical response to CTLA-4 blockade in melanoma. N. Engl. J. Med..

[150] Le D.T., Durham J.N., Smith K.N., Wang H., Bartlett B.R., Aulakh L.K., Lu S., Kemberling H., Wilt C., Luber B.S. (2017). Mismatch repair deficiency predicts response of solid tumors to PD-1 blockade. Science.

[151] Wang S., Xie K., Liu T. (2021). Cancer Immunotherapies: From Efficacy to Resistance Mechanisms—Not Only Checkpoint Matters. Front. Immunol..

[152] Fay E.K., Graff J.N. (2020). Immunotherapy in Prostate Cancer. Cancers.

[153] Emens L.A. (2018). Breast Cancer Immunotherapy: Facts and Hopes. Clin. Cancer Res..

[154] Saleh R., Toor S.M., Khalaf S., Elkord E. (2019). Breast Cancer Cells and PD-1/PD-L1 Blockade Upregulate the Expression of PD-1, CTLA-4, TIM-3 and LAG-3 Immune Checkpoints in CD4(+) T Cells. Vaccines.

[155] Qin S., Xu L., Yi M., Yu S., Wu K., Luo S. (2019). Novel immune checkpoint targets: Moving beyond PD-1 and CTLA-4. Mol. Cancer.

[156] Mohsenzadegan M., Bavandpour P., Nowroozi M.R., Amini E., Kourosh-Arami M., Momeni S.A., Bokaie S., Sharifi L. (2021). The Potential of T Cell Immunoglobulin and Mucin-Domain Containing-3 (Tim-3) in Designing Novel Immunotherapy for Bladder Cancer. Endocr. Metab. Immune Disord. Drug Targets.

[157] Abuodeh Y., Venkat P., Kim S. (2016). Systematic review of case reports on the abscopal effect. Curr. Probl. Cancer.

[158] Janopaul-Naylor J.R., Shen Y., Qian D.C., Buchwald Z.S. (2021). The Abscopal Effect: A Review of Pre-Clinical and Clinical Advances. Int. J. Mol. Sci..

[159] Dewan M.Z., Galloway A.E., Kawashima N., Dewyngaert J.K., Babb J.S., Formenti S.C., Demaria S. (2009). Fractionated but Not Single-Dose Radiotherapy Induces an Immune-Mediated Abscopal Effect when Combined with Anti–CTLA-4 Antibody. Clin. Cancer Res..

[160] Demaria S., Kawashima N., Yang A.M., Devitt M.L., Babb J.S., Allison J.P., Formenti S.C. (2005). Immune-mediated inhibition of metastases after treatment with local radiation and CTLA-4 blockade in a mouse model of breast cancer. Clin. Cancer Res..

[161] Arnold K.M., Flynn N.J., Raben A., Romak L., Yu Y., Dicker A.P., Mourtada F., Sims-Mourtada J. (2018). The Impact of Radiation on the Tumor Microenvironment: Effect of Dose and Fractionation Schedules. Cancer Growth Metastasis.

[162] Rudqvist N.-P., Pilones K.A., Lhuillier C., Wennerberg E., Sidhom J.-W., Emerson R.O., Robins H.S., Schneck J., Formenti S.C., Demaria S. (2018). Radiotherapy and CTLA-4 Blockade Shape the TCR Repertoire of Tumor-Infiltrating T Cells. Cancer Immunol. Res..

[163] Gong X., Li X., Jiang T., Xie H., Zhu Z., Zhou F., Zhou C. (2017). Combined Radiotherapy and Anti–PD-L1 Antibody Synergistically Enhances Antitumor Effect in Non–Small Cell Lung Cancer. J. Thorac. Oncol..

[164] Pilones K.A., Hensler M., Daviaud C., Kraynak J., Fucikova J., Galluzzi L., Demaria S., Formenti S.C. (2020). Converging focal radiation and immunotherapy in a preclinical model of triple negative breast cancer: Contribution of VISTA blockade. Oncoimmunology.

[165] Zeng J., See A.P., Phallen J., Jackson C.M., Belcaid Z., Ruzevick J., Durham N., Meyer C., Harris T.J., Albesiano E. (2013). Anti-PD-1 blockade and stereotactic radiation produce long-term survival in mice with intracranial gliomas. Int. J. Radiat. Oncol. Biol. Phys.

[166] Wei J., Montalvo-Ortiz W., Yu L., Krasco A., Ebstein S., Cortez C., Lowy I., Murphy A.J., Sleeman M.A., Skokos D. (2021). Sequence of αPD-1 relative to local tumor irradiation determines the induction of abscopal antitumor immune responses. Sci. Immunol..

[167] Dovedi S.J., Adlard A.L., Lipowska-Bhalla G., McKenna C., Jones S., Cheadle E.J., Stratford I.J., Poon E., Morrow M., Stewart R. (2014). Acquired Resistance to Fractionated Radiotherapy Can Be Overcome by Concurrent PD-L1 Blockade. Cancer Res..

[168] Young K.H., Baird J.R., Savage T., Cottam B., Friedman D., Bambina S., Messenheimer D.J., Fox B., Newell P., Bahjat K.S. (2016). Optimizing Timing of Immunotherapy Improves Control of Tumors by Hypofractionated Radiation Therapy. PLoS ONE.

[169] Golden E.B., Demaria S., Schiff P.B., Chachoua A., Formenti S.C. (2013). An abscopal response to radiation and ipilimumab in a patient with metastatic non-small cell lung cancer. Cancer Immunol. Res..

[170] Stamell E.F., Wolchok J.D., Gnjatic S., Lee N.Y., Brownell I. (2013). The abscopal effect associated with a systemic anti-melanoma immune response. Int. J. Radiat. Oncol. Biol. Phys..

[171] Postow M.A., Callahan M.K., Barker C.A., Yamada Y., Yuan J., Kitano S., Mu Z., Rasalan T., Adamow M., Ritter E. (2012). Immunologic correlates of the abscopal effect in a patient with melanoma. N. Engl. J. Med..

[172] Chicas-Sett R., Morales-Orue I., Rodriguez-Abreu D., Lara-Jimenez P. (2018). Combining radiotherapy and ipilimumab induces clinically relevant radiation-induced abscopal effects in metastatic melanoma patients: A systematic review. Clin. Transl. Radiat. Oncol..

[173] Koller K.M., Mackley H.B., Liu J., Wagner H., Talamo G., Schell T.D., Pameijer C., Neves R.I., Anderson B., Kokolus K.M. (2017). Improved survival and complete response rates in patients with advanced melanoma treated with concurrent ipilimumab and radiotherapy versus ipilimumab alone. Cancer Biol. Ther..

[174] Formenti S.C., Rudqvist N.P., Golden E., Cooper B., Wennerberg E., Lhuillier C., Vanpouille-Box C., Friedman K., Ferrari de Andrade L., Wucherpfennig K.W. (2018). Radiotherapy induces responses of lung cancer to CTLA-4 blockade. Nat. Med..

[175] Bestvina C.M., Pointer K.B., Karrison T., Al-Hallaq H., Hoffman P.C., Jelinek M.J., Juloori A., Melotek J.M., Murgu S., Partouche J. (2022). A Phase 1 Trial of Concurrent or Sequential Ipilimumab, Nivolumab, and Stereotactic Body Radiotherapy in Patients With Stage IV NSCLC Study. J. Thorac. Oncol..

[176] Antonia S.J., Balmanoukian A., Brahmer J., Ou S.I., Hellmann M.D., Kim S.W., Ahn M.J., Kim D.W., Gutierrez M., Liu S.V. (2019). Clinical Activity, Tolerability, and Long-Term Follow-Up of Durvalumab in Patients With Advanced NSCLC. J. Thorac. Oncol..

[177] Yamaguchi O., Kaira K., Hashimoto K., Mouri A., Miura Y., Shiono A., Nishihara F., Murayama Y., Noda S.E., Kato S. (2019). Radiotherapy is an independent prognostic marker of favorable prognosis in non-small cell lung cancer patients after treatment with the immune checkpoint inhibitor, nivolumab. Thorac. Cancer.

[178] Shaverdian N., Lisberg A.E., Bornazyan K., Veruttipong D., Goldman J.W., Formenti S.C., Garon E.B., Lee P. (2017). Previous radiotherapy and the clinical activity and toxicity of pembrolizumab in the treatment of non-small-cell lung cancer: A secondary analysis of the KEYNOTE-001 phase 1 trial. Lancet Oncol..

[179] De Ruysscher D., Ramalingam S., Urbanic J., Gerber D.E., Tan D.S.W., Cai J., Li A., Peters S. (2022). CheckMate 73L: A Phase 3 Study Comparing Nivolumab Plus Concurrent Chemoradiotherapy Followed by Nivolumab With or Without Ipilimumab Versus Concurrent Chemoradiotherapy Followed by Durvalumab for Previously Untreated, Locally Advanced Stage III Non-Small-Cell Lung Cancer. Clin. Lung Cancer.

[180] Gill S., Nowak A.K., Bowyer S., Endersby R., Ebert M.A., Cook A. (2022). Clinical evidence for synergy between immunotherapy and radiotherapy (SITAR). J. Med. Imaging Radiat. Oncol..

[181] McGranahan N., Furness A.J., Rosenthal R., Ramskov S., Lyngaa R., Saini S.K., Jamal-Hanjani M., Wilson G.A., Birkbak N.J., Hiley C.T. (2016). Clonal neoantigens elicit T cell immunoreactivity and sensitivity to immune checkpoint blockade. Science.

[182] Rizvi N.A., Hellmann M.D., Snyder A., Kvistborg P., Makarov V., Havel J.J., Lee W., Yuan J., Wong P., Ho T.S. (2015). Mutational landscape determines sensitivity to PD-1 blockade in non–small cell lung cancer. Science.

[183] Galon J., Bruni D. (2019). Approaches to treat immune hot, altered and cold tumours with combination immunotherapies. Nat. Rev. Drug Discov..

[184] Higuchi T., Flies D.B., Marjon N.A., Mantia-Smaldone G., Ronner L., Gimotty P.A., Adams S.F. (2015). CTLA-4 Blockade Synergizes Therapeutically with PARP Inhibition in BRCA1-Deficient Ovarian Cancer. Cancer Immunol. Res..

[185] Ding L., Kim H.J., Wang Q., Kearns M., Jiang T., Ohlson C.E., Li B.B., Xie S., Liu J.F., Stover E.H. (2018). PARP Inhibition Elicits STING-Dependent Antitumor Immunity in Brca1-Deficient Ovarian Cancer. Cell Rep..

[186] Shen J., Zhao W., Ju Z., Wang L., Peng Y., Labrie M., Yap T.A., Mills G.B., Peng G. (2019). PARPi Triggers the STING-Dependent Immune Response and Enhances the Therapeutic Efficacy of Immune Checkpoint Blockade Independent of BRCAness. Cancer Res..

[187] Sen T., Rodriguez B.L., Chen L., Corte C.M.D., Morikawa N., Fujimoto J., Cristea S., Nguyen T., Diao L., Li L. (2019). Targeting DNA Damage Response Promotes Antitumor Immunity through STING-Mediated T-cell Activation in Small Cell Lung Cancer. Cancer Discov..

[188] Adams S.F., Rixe O., Lee J.-H., McCance D.J., Westgate S., Eberhardt S.C., Rutledge T., Muller C. (2017). Phase I study combining olaparib and tremelimumab for the treatment of women with BRCA-deficient recurrent ovarian cancer. J. Clin. Oncol..

[189] Lee J.-M., Cimino-Mathews A., Peer C.J., Zimmer A., Lipkowitz S., Annunziata C.M., Cao L., Harrell M.I., Swisher E.M., Houston N. (2017). Safety and Clinical Activity of the Programmed Death-Ligand 1 Inhibitor Durvalumab in Combination With Poly (ADP-Ribose) Polymerase Inhibitor Olaparib or Vascular Endothelial Growth Factor Receptor 1-3 Inhibitor Cediranib in Women’s Cancers: A Dose-Escalation, Phase I Study. J. Clin. Oncol..

[190] Friedlander M., Meniawy T., Markman B., Mileshkin L., Harnett P., Millward M., Lundy J., Freimund A., Norris C., Mu S. (2019). Pamiparib in combination with tislelizumab in patients with advanced solid tumours: Results from the dose-escalation stage of a multicentre, open-label, phase 1a/b trial. Lancet Oncol..

[191] Konstantinopoulos P.A., Waggoner S., Vidal G.A., Mita M., Moroney J.W., Holloway R., Van Le L., Sachdev J.C., Chapman-Davis E., Colon-Otero G. (2019). Single-Arm Phases 1 and 2 Trial of Niraparib in Combination With Pembrolizumab in Patients With Recurrent Platinum-Resistant Ovarian Carcinoma. JAMA Oncol..

[192] Lampert E.J., Zimmer A., Padget M., Cimino-Mathews A., Nair J.R., Liu Y., Swisher E.M., Hodge J.W., Nixon A.B., Nichols E. (2020). Combination of PARP Inhibitor Olaparib, and PD-L1 Inhibitor Durvalumab, in Recurrent Ovarian Cancer: A Proof-of-Concept Phase II Study. Clin. Cancer Res..

[193] Färkkilä A., Gulhan D.C., Casado J., Jacobson C.A., Nguyen H., Kochupurakkal B., Maliga Z., Yapp C., Chen Y.A., Schapiro D. (2020). Immunogenomic profiling determines responses to combined PARP and PD-1 inhibition in ovarian cancer. Nat. Commun..

[194] Zhang N., Gao Y., Huang Z., Dai P., Luo Y., Wu Q., Jiang X., Sun W., Zhang J., Han L. (2022). PARP inhibitor plus radiotherapy reshapes an inflamed tumor microenvironment that sensitizes small cell lung cancer to the anti-PD-1 immunotherapy. Cancer Lett..

[195] Jabbour S.K., Cho B.C., Bria E., Kato T., Bhosle J., Gainor J.F., Reguart N., Wang L., Morgensztern D., Shentu Y. (2022). Rationale and Design of the Phase III KEYLYNK-012 Study of Pembrolizumab and Concurrent Chemoradiotherapy Followed by Pembrolizumab With or Without Olaparib for Stage III Non-Small-Cell Lung Cancer. Clin. Lung Cancer.

